# The Utility of Differential Scanning Calorimetry Curves of Blood Plasma for Diagnosis, Subtype Differentiation and Predicted Survival in Lung Cancer

**DOI:** 10.3390/cancers13215326

**Published:** 2021-10-23

**Authors:** Gabriela Schneider, Alagammai Kaliappan, Taylor Q. Nguyen, Robert Buscaglia, Guy N. Brock, Melissa Barousse Hall, Crissie DeSpirito, Daniel W. Wilkey, Michael L. Merchant, Jon B. Klein, Tanya A. Wiese, Hiram L. Rivas-Perez, Goetz H. Kloecker, Nichola C. Garbett

**Affiliations:** 1 UofL Health—Brown Cancer Center and Division of Medical Oncology and Hematology, Department of Medicine, University of Louisville, Louisville, KY 40202, USA; gabriela.schneider@louisville.edu (G.S.); alagammai.kaliappan@louisville.edu (A.K.); tqng222@uky.edu (T.Q.N.); melissa.hall.2@louisville.edu (M.B.H.); ghkloe01@louisville.edu (G.H.K.); 2 Department of Mathematics and Statistics, Northern Arizona University, Flagstaff, AZ 86011, USA; robert.buscaglia@nau.edu; 3 Department of Biomedical Informatics, College of Medicine, The Ohio State University, Columbus, OH 43210, USA; guy.brock@osumc.edu; 4 Division of Pulmonary, Critical Care and Sleep Disorders Medicine, Department of Medicine, University of Louisville, Louisville, KY 40202, USA; crissie.despirito@louisville.edu (C.D.); Tanya.Wiese@nortonhealthcare.org (T.A.W.); hiram.rivasperez@louisville.edu (H.L.R.-P.); 5 Division of Nephrology and Hypertension, Department of Medicine, University of Louisville, Louisville, KY 40202, USA; daniel.wilkey@louisville.edu (D.W.W.); michael.merchant@louisville.edu (M.L.M.); jon.klein@louisville.edu (J.B.K.); 6 Robley Rex Veterans Affairs Medical Center, Louisville, KY 40202, USA

**Keywords:** differential scanning calorimetry (DSC), DSC curve, diagnosis, overall survival, progression-free survival, lung cancer

## Abstract

**Simple Summary:**

Lung cancer (LC) is the most common malignancy and the leading cause of cancer deaths in the world. Limitations of current screening approaches, such as substantial cost, radiation exposure, and high-false positive rates as well as increasing numbers of LC diagnoses in people without known risk factors, indicate the need for the development of new screening strategies. The aim of our study was to evaluate the utility of differential scanning calorimetry (DSC) for LC patients’ diagnosis. We found that DSC curves could be useful in differentiation of LC patients from control individuals and some changes were subtype or/and stage-dependent. Moreover, some DSC curve features correlated with patients’ overall/progression-free survival. Although the utility of the DSC technique still needs to be confirmed in a clinical setting, with further optimization and development of the classification method, this technique could provide an accurate, non-invasive, radiation-free strategy for LC screening and diagnosis.

**Abstract:**

Early detection of lung cancer (LC) significantly increases the likelihood of successful treatment and improves LC survival rates. Currently, screening (mainly low-dose CT scans) is recommended for individuals at high risk. However, the recent increase in the number of LC cases unrelated to the well-known risk factors, and the high false-positive rate of low-dose CT, indicate a need to develop new, non-invasive methods for LC detection. Therefore, we evaluated the use of differential scanning calorimetry (DSC) for LC patients’ diagnosis and predicted survival. Additionally, by applying mass spectrometry, we investigated whether changes in O- and N-glycosylation of plasma proteins could be an underlying mechanism responsible for observed differences in DSC curves of LC and control subjects. Our results indicate selected DSC curve features could be useful for differentiation of LC patients from controls with some capable of distinction between subtypes and stages of LC. DSC curve features also correlate with LC patients’ overall/progression free survival. Moreover, the development of classification models combining patients’ DSC curves with selected plasma protein glycosylation levels that changed in the presence of LC could improve the sensitivity and specificity of the detection of LC. With further optimization and development of the classification method, DSC could provide an accurate, non-invasive, radiation-free strategy for LC screening and diagnosis.

## 1. Introduction

Lung cancer (LC) is the most common malignancy and the leading cause of cancer deaths in the past few decades. There were an estimated 2.1 million new cases in 2018 worldwide, out of which 234,000 were diagnosed in the United States, representing about 13% of all cancer diagnoses in this country [1]. The 5-year survival rate for LC is 56% for cases detected when the disease is still localized but it drops to only 5% for distant tumors [2]. Unfortunately, only 16% of cases are diagnosed at an early stage; thus, it is estimated that more than half of people with LC die within 1 year of being diagnosed [2].

Smoking, the main cause of small cell lung cancer (SCLC) and non-small cell lung cancer (NSCLC), contributes to 80 and 90% of LC deaths in women and men, respectively [3,4]. Other factors contributing to the incidence of LC include exposure to radon, asbestos, and outdoor air pollution [3]. Screening (mainly using low-dose CT scans) for individuals at high risk has the potential to dramatically improve lung cancer survival rates by diagnosing the disease at an earlier stage [5]. However, in recent years there has been an increase in the number of LC cases unrelated to the well-known risk factors, thus falling outside current screening guidelines and delaying prompt diagnosis and treatment [6]. Moreover, low-dose CT is an expensive method that carries the risk of radiation exposure, and because of its high false-positive rate, could also result in increased risks to patients from invasive follow-up procedures [7]. As a result of these limitations, low-dose CT screening has not been widely implemented outside the United States, and there is a need to develop a method that allows for low-cost and low-risk LC diagnosis.

One potential method for screening and diagnosis of LC is differential scanning calorimetry (DSC). DSC curves reflect the heat change (excess specific heat capacity) in a fluid sample as it is heated, corresponding to the structural changes in the molecular constituents of the fluid as a function of temperature (e.g., protein denaturation). DSC curves have been shown to be a successful tool used in the diagnosis and characterization (e.g., progression or severity) of several cancers, including cervical cancer, breast cancer, colorectal cancer, multiple myeloma, and brain tumors [8,9,10,11,12,13,14]. In the current work, we evaluated the use of selected summary metrics and principal components (PCs) calculated from DSC curves for diagnosis and characterization of LC patients. Moreover, we evaluated their use as a potential predictor of progression-free and overall survival of patients.

One of the current challenges of the development of DSC as a clinical diagnostic technology is the high dimensionality of the acquired data. Unlike most biomarkers for which one parameter is measured and interpreted, DSC analysis results in hundreds of data points, creating a curve where each point carries diagnostic information about the patient sample. In our analysis, we used DSC curve features such as the position and height of the peaks or the total area under the DSC curve. Those summary metrics were further analyzed using widely accepted statistical methods. This approach, unfortunately, does not allow us to evaluate the complete information carried by the DSC curve and requires the development of more complex mathematical and statistical approaches. Several groups are working on the development of algorithms incorporating information from the full DSC curve and simplifying data interpretation. Such development would be beneficial for moving the DSC technique from research laboratories to the clinical setting by distilling several complex parameters into a single diagnostic metric that can be utilized by physicians.

It has been hypothesized that differences between DSC curves reflect alterations in plasma composition resulting from the up- or down-regulation of proteins, the presence of disease-related biomarkers that interact with or covalently modify plasma proteins, or differences in post-translational modifications (PTMs) of the proteins [15]. These changes could affect the thermal stability of plasma proteins and thus alter DSC curves. In cancers, several different PTMs, including glycosylation, are associated with cancer development [16,17]. It has been found that aberrant protein glycosylation, changes in glycoprotein concentrations, or alteration in glycan structures can be used for cancer detection and can be associated with tumor stage and prognosis [16,17,18,19]. In this study, we evaluated differences in the glycosylation level of plasma proteins in specimens from LC patients and control subjects with benign nodules to evaluate whether differences in these PTMs can be related to differences in DSC curves. Moreover, we assessed whether glycosylation levels of selected plasma proteins could be used as biomarkers for LC diagnosis and characterization, such as for the differentiation of LC subtype or stage.

## 2. Materials and Methods

### 2.1. Patient Population

De-identified plasma specimens and pertinent patient data were obtained from lung cancer patients and from benign nodule control patients attending Surgical Oncology, Thoracic Oncology and Pulmonology clinics at the University of Louisville. The study was reviewed and approved by the Institutional Review Board at the University of Louisville (IRB# 15.0374, 15.0508, 08.0388) in compliance with the Helsinki Agreement. Patient samples were deidentified and coded allowing for blinded and unbiased data collection. Patient diagnosis, clinical information, and demographic status were provided for data analysis. All plasma samples were obtained by collecting blood specimens into sodium heparin anticoagulant vacutainers. After isolation, plasma was aliquoted and immediately stored at −80 °C until analysis.

A total of 396 plasma specimens were obtained from 51 control donors and 175 lung cancer patients. Only one specimen per control donor was collected (total of 51 control specimens), whereas one or more longitudinal specimens were collected from LC patients (total of 345 LC specimens). The control group consisted of patients undergoing surveillance for biopsy-, image-, or time-proven benign masses in their lungs who had no active lung or other cancer. Out of this group, 12 patients had a history of cancer other than lung and their inactive cancer diagnosis was established at least 3 years before sample collection, and for 6 of them, more than 10 years before. Out of the total of 345 LC specimens, 38 specimens obtained from 18 lung cancer patients who had active or past diagnoses of non-lung cancer malignancies were excluded from analysis, leaving a total sample set of 307 LC specimens from 157 LC patients. 

For baseline analysis, in which we evaluated LC-related changes in DSC curves not affected by patient treatment, 148 specimens from a total pool of 307 LC specimens were selected based on the following criteria: (1) specimens were collected as close as possible to the diagnosis date, (2) specimens could not be collected during chemotherapy or within 30 days from the end of chemotherapy treatment, and (3) no more than one specimen per patient could be selected. 

Key demographic and clinical factors of the clinical groups included in the study are detailed in Table 1 and Table S1. Of note, our control group consists of 82% of current or ex-smokers. Although the smoking status of the control subjects does not reflect the general population of the United States, both control and LC patients were enrolled from the local patient population attending the University of Louisville clinics, and approximately matched the smoking status of LC patients (94% of current or ex-smokers), thus allowing an examination of DSC curve changes related to lung cancer and not smoking per se.

The table shows patient counts and the percentage of patients within each class. Legend: AC, Adenocarcinoma; NOS, not otherwise specified; NSCLC, non-small cell lung cancer; SCC, squamous cell carcinoma; SCLC, small cell lung cancer.

### 2.2. DSC Sample Preparation

Specimens were prepared as previously described [9]. Before analysis, all plasma specimens (200 μL) were dialyzed for 24 h at 4 °C against a buffer consisting of 1.7 mM KH_2_PO_4_, 8.3 mM K_2_HPO_4_, 150 mM NaCl, 15 mM sodium citrate, and pH 7.5. After dialysis, samples were filtered through 0.45 µm filters to remove particulates. Filtered (0.2 µm) buffer from the final dialysis period was used for sample dilution and as a reference solution for DSC analysis.

### 2.3. Collection of DSC Curves

DSC data were collected as described [9] using a Nano DSC Autosampler System (TA Instruments, New Castle, DE, USA) serviced according to procedures provided by the manufacturer. Additionally, instrument performance was evaluated using lysozyme as a biological standard to confirm that the instrument was within manufacturer’s specifications. Before analysis, dialyzed plasma samples were diluted 25-fold to obtain a total protein concentration ~2 mg/mL. The exact protein concentration was determined for all samples using the bicinchoninic acid protein assay kit according to the manufacture microplate protocol (Pierce, Rockford, IL, USA), using absorbance measurements taken with a Tecan Spark plate reader (Tecan U.S., Research Triangle Park, NC, USA). Next, samples and dialysis buffer were loaded into 96-well plates and placed in the DSC instrument autosampler at 4 °C until analysis. Each sample analysis included a pre-scan equilibration step of 15 min at 20 °C, followed by data collection during heating of the samples from 20 °C to 110 °C at a scan rate of 1 °C/min.

We previously published a methodological study evaluating multiple pre-analytical and analytical variables associated with the handling and analysis of blood plasma specimens, including the effect of sample storage temperature and freeze-thaw cycles, buffer exchange, filtering and sample dilution, and instrument scan replicates [20]. This study showed that DSC curves are unaffected by sample storage at −80 °C and freezing and thawing, or by dialysis, filtration, and dilution prior to DSC analysis, enabling us to develop a standard protocol for sample handling. Importantly, the ability to store samples at −80 °C provides flexibility in the analysis of previously collected specimens in biorepositories and to batch samples for analysis. Our protocol involves the collection of duplicate scans for each sample and batching of samples to ensure that DSC data collection is completed within 7 days from the initial thawing of the samples. Additionally, we evaluated buffer scans before and after the collection of sample DSC curve data to determine reproducibility and effectiveness of instrument chamber cleaning procedures. We also compared sample scans collected after a buffer or sample scan and found it is possible to collect consecutive sample scans after extensive rinsing of the instrument chambers with little effect on the DSC curve. We included example data in Figure S1, showing two complete data sets collected at the beginning and end of the study, demonstrating the reproducibility of duplicate DSC curves. The majority of the difference between repeated measurements of the same sample is observed for the major irreversible denaturation/aggregation effects above 100 °C, whereas almost no difference is observed between duplicate measurements within the 45–90 °C range used for analysis.

DSC data were post-processed using Origin 7 (OriginLab Corporation, Northampton, MA, USA). First, we corrected the raw data for instrumental baseline by subtraction of a suitable buffer scan. Next, DSC curve data were normalized for total protein concentration and corrected for non-zero baselines by application of a linear baseline fit. All data are presented as the average of duplicate measurements and plotted as excess specific heat capacity (cal/°C.g) versus temperature (°C). DSC curve data for all patient samples included in this study can be found in Table S2.

### 2.4. Mass Spectrometry

The Global O- and N-linked protein glycosylation levels were analyzed using liquid chromatography-mass spectrometry (LCMS)-based glycoproteomic approach. Of the 51 control specimens and 148 LC specimens identified for baseline DSC curve analysis, 124 specimens were selected for LCMS analysis. Eight of the 124 samples were flagged for low data quality (plasma coloration suggesting red blood cell hemolysis) and were removed from subsequent data analysis. Detailed information about specimens that were included for mass spectrometry analysis is shown in Table S1.

#### 2.4.1. Sample Digestion

Plasma sample protein concentrations were estimated after dilution into liquid chromatography mass spectrometry (LC-MS) grade water (Thermo Fisher Scientific, Waltham, MA, USA) by Bradford protein assay (BioRad, Hercules, CA, USA) against a bovine serum albumin concentration curve. Protein samples (20 µg) were dried, resuspended in a 25:1:2 ratio mixture of 1M triethylammonium bicarbonate (TEA-BC, Millipore Sigma, St. Louis, MO, USA) pH 8.5, 2% SDS, and 50 mM tris(2-carboxyethyl)phosphine (TCEP, Thermo Fisher Scientific), and heated to 60 °C for 1 h. The reduced samples were cooled to room temperature, centrifuged, and mixed with 10 µL 3.36 mM iodoacetamide prior to incubation in the dark for 30 min. Alkylated protein samples were digested using 0.4 µg sequencing grade modified trypsin (Promega, Madison, WI, USA) for a 1:50 enzyme:protein ratio at 37 °C overnight. This process was repeated using a pooled (440 µg) plasma protein sample to be used as an internal standard channel. Digested samples were stored at −80 °C.

#### 2.4.2. Tandem Mass Tag Labeling

Digested plasma samples were labeled with tandem mass tag (TMT) 10-plex labeling reagents (Thermo Fisher Scientific) according to the manufacturer’s guidelines with some modifications. The digested internal standard was labeled with the TMT 10-plex reagent TMT-131. In brief, TMT vials were warmed, anhydrous acetonitrile was added, the vial vortexed occasionally over 5 min, and then centrifuged to collect reagent. TMT reagent (0.2 unit) was transferred to the sample vial and incubated for 1h. A second aliquot (0.2 unit) was added and incubated for 3 h. An aliquot (3.2 μL) of 5% NH_2_OH was incubated with the sample for 15 min to quench the excess labeling reagent. Individual flights of nine samples (5 μg per channel) plus internal standard channel (TMT-131 label) were admixed and stored at −80 °C prior to the next step for glycopeptide isolation.

#### 2.4.3. Deglycosylation of the TMT Sample Flights

Digested, TMT 10-plex labeled samples were thawed and diluted with 50 mM Na_2_HPO_4_ pH 7.5 so that acetonitrile has a final concentration of 2% *v*/*v*. Microcon-10 (YM-10) spin concentrators housing regenerated cellulose filters (10,000NMWL Ultracel, Millipore Sigma) were used for sample concentration and buffer exchange. Volumes were reduced by centrifugation at 14,000× *g* and 20 °C. The first sample ultra-filtrate was collected in a tube and stored at −80 °C. Sample retained on the YM-10 filter was rinsed three times with 100 µL 50 mM Na_2_HPO_4_ pH 7.5 and the YM-10 insert transferred to a new receiving tube. The sample was resuspended in 50mM Na_2_HPO_4_ pH7.5 and digested using 2.5 µL of the Protein Deglycosylation Mix II (New England BioLabs, Ipswich, MA, USA) using the non-denaturing protocol provided by the manufacturer. The sample was agitated for mixing and incubated at 37 °C overnight. The deglycosylated peptides were collected by centrifugation at 14,000× *g* and 20 °C for 30 min. The filter was rinsed with 50 uL 0.3 M NaCl and collected at 14,000× *g* and 20 °C for 20 min. The ultra-filtrates were pooled, adjusted to a final concentration of 2% (*v*/*v*) acetonitrile/0.4% formic acid for clean-up using C18 PROTOTM, 300 Å Ultra MicroSpin columns (Nest Group, Southborough, MA, USA).

#### 2.4.4. Liquid Chromatography

Proteomic data were collected on the deglycosylated peptide samples as previously described [21]. Peptides were loaded onto an Acclaim PepMap 100 75 µm × 2 cm, nanoViper (C18, 3 µm, 100 Å) trap column prior to separation at 250 nL/min on an Acclaim PepMap RSLC 75 µm × 50 cm, nanoViper (C18, 2 µm, 100 Å) separating column (Thermo Fisher Scientific) at 50 °C with a 165 min 2–41% acetonitrile/0.1% formic acid gradient using an EASY-nLC 1000 UHPLC system (Thermo Fisher Scientific). Eluted peptides were introduced into the Orbitrap ELITE mass spectrometer using a Nanospray Flex source (Thermo Fisher Scientific) with an ion transfer capillary temperature of 225 °C and 1.75 kV spray voltage. A 40 mm stainless steel emitter (Thermo Fisher Scientific) was coupled to the outlet of the separating column.

#### 2.4.5. Data Acquisition

An Orbitrap Elite ETD mass spectrometer (Thermo Fisher Scientific) was used to collect data. An Nth Order Double Play method was created in Xcalibur v2.2 (Thermo Fisher Scientific). Scan event one of the method obtained an FTMS MS1 scan (normal mass range; 60,000 resolution, full scan type, positive polarity, centroid data type) for the range 300–2000 *m*/*z*. Scan event two obtained FTMS HCD MS2 scans (normal mass range; 60,000 resolution; centroid data type) on up to 10 peaks that had a minimum signal threshold of 5000. The lock mass option was enabled (0% lock mass abundance) using the 371.101236 *m*/*z* polysiloxane peak as an internal calibrant.

#### 2.4.6. Data Analysis with Proteome Discoverer v2.1.1.21

Proteome Discoverer v2.1.1.21 (Thermo Fisher Scientific) was used to analyze the RAW data collected by the mass spectrometer. The database used in Mascot v2.5.1 (Matrix Science Inc., Boston, MA, USA) and SequestHT (Thermo Fisher Scientific) searches was the 1/18/2018 version of the UniprotKB Homo sapiens reviewed proteome canonical and isoform sequences. Search criteria included up to two missed tryptic cleavages, minimal length of six amino acids, 10 ppm precursor and 0.02 Da fragment mass tolerances, cysteine carbamidomethylation as static and methionine oxidation, lysine or N-terminal TMT 10plex, and asparagine deamidation (e.g., deglycosylation) as dynamic modifications. A Percolator node was included in the Proteome Discoverer workflow to correct for multiple comparisons with a maximum delta correlation (delta Cn) of 0.05 and maximum rank of 0 (no restriction) for input data. A decoy database strategy was used with target false discovery rates (FDR) set to 0.01 for strict and 0.05 for relaxed q-value controls.

### 2.5. Statistical Analysis of DSC Curves

**Figure 1:**
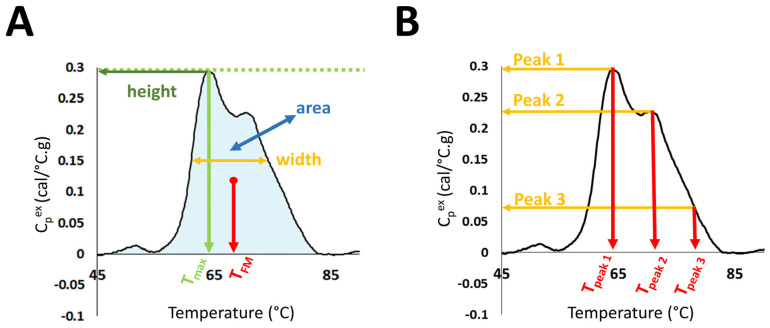
Selected DSC curve features evaluated in the study. (**A**) DSC curve peak width at half height; total area under the curve, maximum peak height; temperature of the peak maximum (T_max_); and first moment temperature, T_FM_. (**B**) Maximum excess specific heat capacity (C_p_^ex^) and position of Peaks 1, 2, and 3.

Evaluation of DSC curves was performed in the temperature range 45–90 °C through the calculation of several summary metrics: DSC curve peak width at half height; total area under the curve; maximum peak height; temperature of the peak maximum (T_max_); maximum excess specific heat capacity (C_p_^ex^) of the first peak in the region 60–66.9 °C (Peak 1); maximum C_p_^ex^ of the second peak in the region 67–72.9 °C (Peak 2); maximum C_p_^ex^ of the third peak in the region 73–78 °C (Peak 3); the position of Peak1 (T_peak 1_), Peak 2 (T_peak 2_), and Peak 3 (T_peak 3_); as well as Peak 1/Peak 2 ratio (Peak 1/2); Peak 1/Peak 3 ratio (Peak 1/3); Peak 2/Peak 3 ratio (Peak 2/3); and the first moment temperature, T_FM_ (Figure 1).

T_FM_ was calculated using Equation (1).

(1) $$T_{\mathrm{FM}}=\frac{\int_{45}^{90}\left(TC_{p}^{ex}\right)dT}{\int_{45}^{90}C_{p}^{ex}dT}$$

Intuitively, the T_FM_ corresponds to a central mass point when considering the DSC curve as a density curve. In addition, to use the full information from the DSC curve, we calculated principal components (PCs). The utility of DSC curve summary metrics and PCs for patient classification was illustrated via receiver operating characteristic (ROC) curves calculated for each parameter individually. All analyses were done using R software [22].

GraphPad Prism 8 software was used for conducting statistical tests (GraphPad, La Jolla, CA, USA). As T_peak 3_ values were identical in more than 90% of specimens, this metric was excluded from statistical analyses. Normality of each summary metric and PC distribution was tested using the D’Agostino-Pearson omnibus K2 test at the 5% significance level. Since 15 out of 19 analyzed parameters did not pass the normality test, the two-group contrasts of summary metrics and PCs were tested for statistical significance by the Mann–Whitney test. Differences in summary metrics and PCs between three or more groups were tested for statistical significance by one-way Welch’s ANOVA test, followed by the Dunnett T3 comparisons test.

Data were visualized using boxplots with whiskers indicating the 5th and 95th percentiles, and points above or below the whiskers mark the values outside of this range. Analysis of association between the values of summary metrics or PC components and study groups was performed using Fisher’s exact tests. The Mantel-Cox test was used to analyze overall survival and progression-free survival. Glycosylation data were tested for statistical significance by repeated-measures one-way ANOVA with Geisser-Greenhouse correction, followed by Tukey’s multiple comparison test. Statistical analysis was performed using the median values of peptides for each group, whereas values normalized to control data were used for visualization of differences between groups.

All statistical tests run over a family of parameters were adjusted for multiple comparison by applying the false discovery rate method using the *p.adjust* function in R (Table 2 and Table 3 and Tables S3–S5). *p*-value adjustments are required to enforce a family-wide type-I error rate without inflation due to multiple comparisons [23]. Given the large number of parameters that are considered in this study, both unadjusted and adjusted *p*-values are presented in the tables for readers unfamiliar with the impact of false discovery rate adjustment on *p*-values when multiple parameters (here, a total of 19 parameters) are evaluated in a study. Only adjusted *p*-values are presented on graphs. Although our primary consideration of differences between sample sets was at the 5% significance level, we also evaluated results at less stringent type-I error rates.

## 3. Results

Over the last ~15 years, the focus of multiple research groups on the development of DSC as a clinical diagnostic technology has evolved from small, proof-of-principle studies to larger studies aimed at exploring relationships between DSC curve parameters and a given clinical condition. Our motivation in this study was to rigorously examine DSC curve differences between clinical groups through the evaluation of a large number of parameters and to examine the relationships of these parameters to multiple clinical characteristics of LC. We evaluated a total of 19 parameters: 13 summary metrics corresponding to localized features of the DSC curve (Figure 1) and 6 PCs. These parameters were compared between all LC patients, as well as by stage and subtype of LC. We also considered smoking-related differences between LC patients and controls, and the relationship of DSC curve parameters to overall and progression-free survival of patients. Finally, we examined the global O- and N-linked protein glycosylation levels in patient samples as a possible protein modification that could affect DSC curve features.

Given the large number of DSC curve and clinical parameters examined, only the most impactful results were included in the main manuscript, with all additional data provided in the supplementary materials. For a general audience that may not be familiar with statistical tests, one important point to note here is that evaluating 19 DSC curve parameters requires adjusting *p*-values for multiple comparisons by applying the false discovery rate method. To show the effect of this adjustment we presented both unadjusted and adjusted *p*-values in tables, but only report adjusted *p*-values on graphs. In presenting the results, we discuss results primarily at the 5% significance level but use less stringent type-I error rates in the presence of large adjustments. Although many researchers focus on the 5% significance level, we did not want to restrict our analysis to this level given that other published studies evaluate fewer parameters that would be subject to less FDR adjustment. Moreover, parameters that are slightly above the 5% level may become impactful in a larger study. To motivate additional investigation of our study data and promote further development of the clinical DSC field, we are providing all DSC curve and clinical data files as part of the supplementary material.

### 3.1. Comparison of DSC Curves between LC Patients and Controls

**Figure 2:**
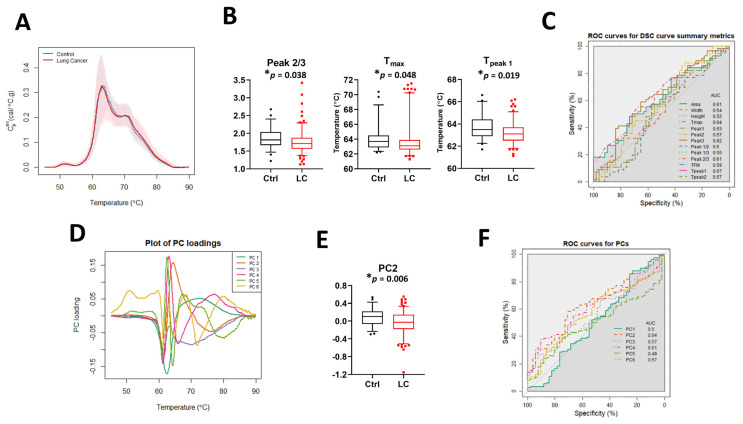
The potential utility of summary metrics and principal components to differentiate LC patients from controls. (**A**) Plot of the median DSC curve value at each temperature for LC and control subjects. Bands represent the 5th and 95th percentiles among subjects at each temperature. (**B**) Boxplots of selected summary metrics calculated from DSC curves for LC patients and controls. (**C**) Classification performance of the summary metrics determined by receiver operating characteristic (ROC) curves. (**D**) Plot of selected PC loadings at each temperature. (**E**) Boxplots of PC2 values calculated from DSC curves for LC patients and controls. (**F**) Classification performance of PCs as determined by receiver operating characteristic (ROC) curves. *, adjusted *p*-value < 0.05.

The median DSC curves for baseline LC patients and controls (Ctrl) revealed no visual differences between the two sets of subjects (Figure 2A). Examination of DSC curve differences between clinical groups was first performed by calculation of the 13 DSC curve summary metrics (Figure 2B and Figure S2A), excluding T_peak 3_ because its value was identical (75.5 °C) in more than 90% of specimens. Statistically significant differences between controls and LC patients were observed for Peak 2/3, T_max,_ and T_peak 1_. The classification performance of each metric was assessed using the area under the curve (AUC) of ROC curves (Figure 2C). The results showed that individual summary metrics had modest AUC values; the highest observed AUC was 0.67 for T_peak 1_. Principal component analysis (Figure 2D,E and Figure S2B,C) revealed a statistically significant difference for PC2 between controls and LC patients but the AUC value (Figure 2F) showed that this PC was not particularly predictive (AUC = 0.64). Overall, these results suggest that neither a single summary metric nor PC component is useful for the classification of LC and control patients.

To analyze whether there is an association between summary metrics or PCs and the study group, we compared their distribution in controls and LC patients when compared to the median value and interquartile range (IQR) calculated for 51 control specimens (Table 2 and Table S4). *p*-value adjustments were made separately for two families of metrics: those partitioned based on the median value calculated for all control specimens and metrics partitioned based on the IQR calculated for the control group. Although after adjustment we did not observe differences between the two families of metrics at the 5% significance level, our results suggest that there are some trends that should be further investigated when more DSC data will become available. For example, we observed differences in unadjusted *p*-values at the 5% significance level between LC patients and control subjects in the distribution of the values for T_max_, T_peak 1_, T_peak 2_, PC2, and PC5 (Table 2). Interestingly, we also noticed that almost 63% of T_peak 2_ values and more than 70% of PC5 values for LC patient DSC curves were localized outside the IQR, showing the heterogeneity of DSC curves within the LC group compared to controls.

**Table 2:** Selected associations between summary metrics/PCs and the clinical group. Summary metrics/PCs were dichotomized based on either the median value or interquartile range (IQR) calculated for control subjects.

Parameter	Range	Control (*n* = 51)	LC (*n* = 148)	Unadjusted *p*-Value	FDR ^1^ Adjusted *p*-Value
T_max_	<median	25 (49.0%)	99 (66.9%)	* 0.0293	0.1856
	≥median	26 (51.0%)	49 (33.1%)		
T_peak1_	<median	23 (45.1%)	99 (66.9%)	* 0.0076	0.1444
	≥median	28 (54.9%)	49 (33.1%)		
T_peak2_	Outside IQR	23 (45.1%)	93 (62.8%)	* 0.0325	0.3088
	Within IQR	28 (54.9%)	55 (37.2%)		
PC2	<median	25 (49.0%)	100 (67.6%)	* 0.0283	0.1856
	≥median	26 (51.0%)	48 (32.4%)		
PC5	Outside IQR	26 (51.0%)	105 (70.9%)	* 0.0159	0.3021
	Within IQR	25 (49.0%)	43 (29.1%)		

Legend: ^1^ FDR, false discovery rate; * unadjusted *p*-value < 0.05; IQR, interquartile range [Q1, Q3].

### 3.2. Smoking-Related Differences between LC and Control Patients

Given the strong association between smoking and lung cancer cases, we examined if there was a difference between DSC curves of LC and control patients when separated by smoking status: current smokers (CS), ex-smokers (ExS), and never smokers (NS; Figure S3 and Table S3). Similar to our observations of the distribution of DSC curve parameters between LC patients and controls, we did not observe differences at the 5% significance level after adjustment. This may reflect the imbalance in smoking status between groups (e.g., only 29% of current smokers in the control group vs. 43% in the LC group). However, we did observe smoking-related differences at less stringent significance levels for T_peak 1_ (*p*-value = 0.014, adj. *p*-value = 0.199), PC2 (*p*-value = 0.009, adj. *p*-value = 0.174), and PC4 (*p*-value = 0.018, adj. *p*-value = 0.174). These parameters may be of interest for patient differentiation with the availability of additional DSC data from lung cancer patients.

### 3.3. Impact of Stage and Type of Cancer on DSC Curves

**Figure 3:**
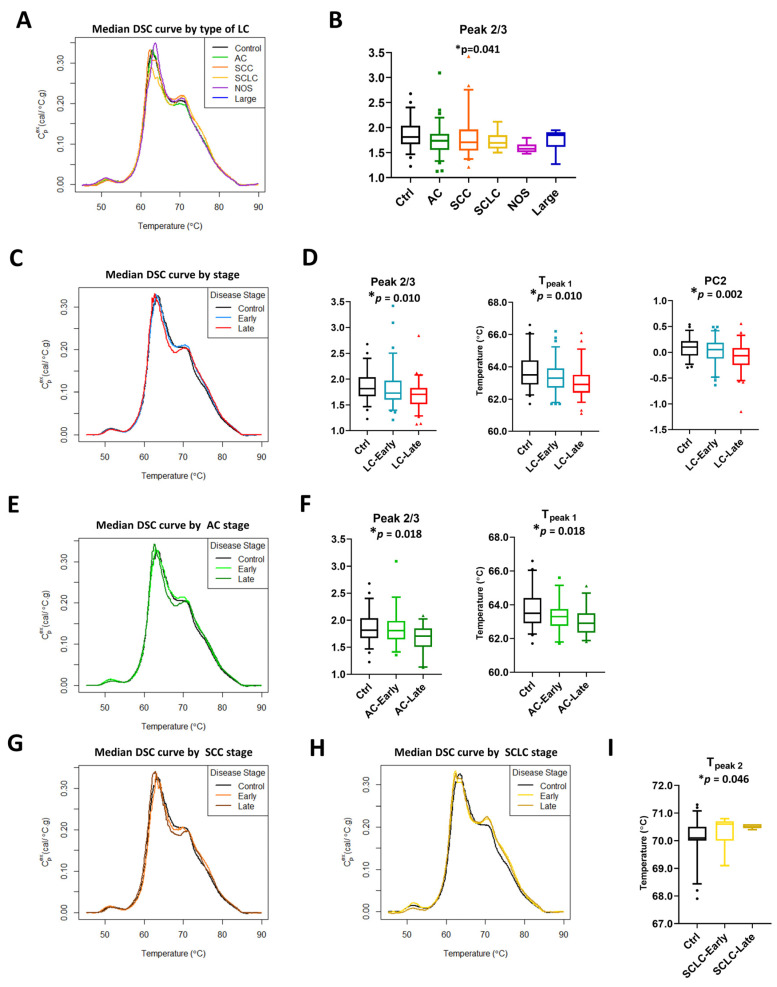
Subtypes and stage-related differences in DSC curves of LC specimens. (**A**) Plot of the median DSC curve value at each temperature for different subtypes of LC and control subjects. (**B**) Boxplots of selected summary metrics calculated from DSC curves for controls and different subtypes of LC patients. Plot of the median DSC curve value at each temperature for control subjects and early stage (stage 1–3a) or late stage (stage 3b–4) LC (**C**), AC (**E**), SCC (**G**), and SCLC (**H**) patients. Boxplots of selected summary metrics and PCs calculated from DSC curves for controls and early/late stage LC (**D**), AC (**F**), and SCLC (**I**) patients. *, adjusted *p*-value < 0.05.

The lung cancer study group consisted of patients with different LC subtypes and stages. To examine their effect on DSC curve summary metrics and calculated PCs, patients were separated by type and stage of lung cancer (Figure 3 and Figures S4–S8). The graphical display of median DSC curves of different subtypes of LC patients compared with controls revealed some visual differences between the clinical groups (Figure 3A). However, statistically significant differences between groups were observed only for Peak 2/3 (Figure 3B). Similarly, we observed some visual differences between DSC curves for patients diagnosed with late stage LC (stages 3b–4 in NSCLC and extensive stage in SCLC; Ext) and early stage LC (stages 1–3a in NSCLC and limited stage in SCLC; Lim) or those with benign lung masses (controls). Significant differences were found for Peak 2/3 and T_peak 1_ (Figure 3C,D, Figure S5A,B). Additionally, PC analysis revealed that PC2 is statistically different between control and LC specimens divided by stage (Figure 3D).

To examine if DSC curves were sensitive to stage within each subtype, we performed stage-dependent analysis of summary metrics and PCs for LC patients within adenocarcinoma (AC, Figure 3E,F and Figure S6), squamous cell carcinoma (SCC, Figure 3G, Figure S7), and SCLC (Figure 3H,I, Figure S8) subtypes. Other subtypes of lung cancer were excluded from stage-dependent analysis because of limited sample sizes (not otherwise specified, NOS, *n* = 8, and large cell carcinoma, Large, *n* = 5). Minor visual differences in DSC curves between early stage/controls and late stage within a particular LC subtype were observed (Figure 3E,G,H). Analysis showed that Peak 2/3 and T_peak 1_ were different between AC groups, with the observation that their values decreased in a stage-dependent manner (Figure 3F). In SCLC, we observed an increase in T_peak 2_ values between control subjects, early, and late stage patients with a highly statistically significant difference between the groups (Figure 3I). In contrast to AC and SCLC, no differences based on adjusted *p*-values were detected at the 5% level in SCC, however, our analysis indicated additional parameters were significant at 10%, including T_max_ (*p*-value = 0.009, adj. *p*-value = 0.089) and T_peak 1_ (*p*-value = 0.014, adj. *p*-value = 0.091; Figure S7 and Table S3), and may be of interest to investigate in future studies.

Primary PC analysis was consistent with our previous observations, showing that before adjustments, PC2 was significantly different between lung cancer stage for AC (*p*-value = 0.014; Table S3) and SCC (*p*-value = 0.009; Table S3) but required a less stringent significance level of 10% after accounting for multiple comparisons (adj. *p*-value = 0.088 and 0.089, respectively; Figures S6 and S7, Table S3). Taken together, our results indicate the sensitivity of DSC curves to different subtypes and stages of lung cancer, with differences in multiple summary metrics and PC values that may be of potential utility for the differentiation of clinical groups.

### 3.4. Glycosylation as a Possible Post-Translational Modification Affecting DSC Curves

**Figure 4:**
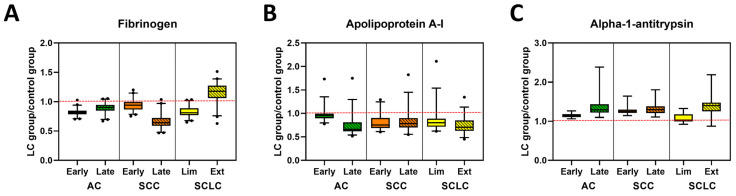
Glycosylation profiles of selected plasma proteins in control and lung cancer patient specimens. Examples of glycosylation levels of selected proteins involved in coagulation (**A**), transportation (**B**) or belonging to the serpins family (**C**) in patients with different stages of AC, SCC, and SCLC. Data are presented normalized to control values, (LC group)/(Control group). The dashed red line indicates the median value of control samples.

PTMs might be responsible for changes in DSC curves observed for LC patients. One such modification is the glycosylation of proteins. Thus, we examined the glycosylation level in specimens from LC patients compared with control subjects. For analysis, only data for which a particular peptide was detected in at least 50% of the specimens within each clinical group was used. Median values for each group were calculated and a “heat map” constructed where the highest median value for a particular peptide was marked in red, and the lowest was marked in blue (Figure S9). Peptides were grouped based on the functions of the proteins to which they relate. Additionally, we combined the data for all peptides corresponding to a single protein (with the exception of immunoglobulins where peptide data corresponding to different immunoglobulin chains were combined) and evaluated the statistical significance of differences in the level of protein glycosylation between LC groups and control subjects (Table 3 and Table S5). For visualization of differences in the total glycosylation of a selected protein, these data were recalculated as fold difference of LC groups relative to control specimens (Figure 4 and Figure S10). Results obtained for late stage SCLC should be treated with caution given the low sample size for this group, although the interesting trends can be pursued in future studies with larger patient numbers. We found only small changes in glycosylation of immunoglobulins when comparing controls with LC, where increased glycosylation level was primarily associated with early stage AC (adj. *p*-value = 0.0026), SCC (adj. *p*-value < 0.0001), and SCLC (adj. *p*-value < 0.0001; Figures S9A and S10A). Moreover, although the glycosylation level of immunoglobulins in late stage SCC specimens was similar to control specimens, it was statistically different from all LC groups (Table S5).

Interestingly, the glycosylation level of C3, a protein that belongs to the complement cascade (Figures S9B and S10A, Table S5) was higher for late stage SCC (adj. *p*-value = 0.0205) and in both stages of SCLC subtypes (adj. *p*-value = 0.0005 and 0.0004, respectively) when compared with controls. In contrast, we did not observe any differences in glycosylation of C4 between all analyzed groups (Figures S9B and S10A, Table S5). When analyzing the glycosylation level of alpha-2-macroglobulin, fibrinogen, and beta-2glycoprotein 1, proteins that are part of the coagulation cascade, we found a stage-dependent decrease in glycosylation level of alpha-2-macroglobulin in AC and SCC (Figure 4A, Figures S9C and S10B, Table S5). Statistical analysis revealed that the late stage AC specimens were significantly different from all other groups except late stage SCC (Table 3 and Table S5). Additionally, glycosylation of alpha-2-macroglobulin for both early and late stages of SCLC was statistically different from those observed for control (adj. *p*-value = 0.0109 and 0.0250, respectively) and late stage SCC (adj. *p*-value = 0.0005 and 0.0120, respectively). High variation in glycosylation levels between subtypes of LC specimens and control were observed for fibrinogen, which was found to be statistically significant between all groups except late stage AC compared with early stage SCC (Figure 4A, Figure S9C and Table S3). In contrast, there were almost no differences between patient groups in the glycosylation level of beta-2 glycoprotein 1 (Table S5).

**Table 3:** Summary of the *p*-values of selected plasma proteins for repeated measures ANOVA and Tukey’s multiple comparisons. Tests evaluate the differences between clinical groups using mass spectrometry glycosylation data of peptides grouped according to protein function.

**FIBRINOGEN**								
**ANOVA Summary**			Unadjusted *p*-Value			FDR ^1^ Adjusted *p*-Value		
			<0.0001			0.0002		
**Tukey’s multiple comparison tests**								
		Control	AC		SCC		SCLC	
			Early	Late	Early	Late	Lim	Ext
Control		X	<0.0001	<0.0001	0.0017	<0.0001	<0.0001	<0.0001
AC	Early	<0.0001	X	0.0011	<0.0001	<0.0001	ns	<0.0001
	Late	<0.0001	0.011	X	ns	<0.0001	0.0107	<0.0001
SCC	Early	0.0017	<0.0001	ns	X	<0.0001	<0.0001	<0.0001
	Late	<0.0001	<0.0001	<0.0001	<0.0001	X	<0.0001	<0.0001
SCLC	Lim	<0.0001	ns	0.0107	<0.0001	<0.0001	X	<0.0001
	Ext	<0.0001	<0.0001	<0.0001	<0.0001	<0.0001	<0.0001	X
**APOLIPOPROTEIN A-I**								
**ANOVA Summary**			Unadjusted *p*-Value			FDR ^1^ Adjusted *p*-Value		
			<0.0001			0.0002		
**Tukey’s multiple comparison tests**								
		Control	AC		SCC		SCLC	
			Early	Late	Early	Late	Lim	Ext
Control		X	0.0214	<0.0001	<0.0001	0.0001	0.0002	<0.0001
AC	Early	0.0214	X	<0.0001	<0.0001	0.0007	0.0004	<0.0001
	Late	<0.0001	<0.0001	X	0.0169	0.0214	<0.0001	ns
SCC	Early	<0.0001	<0.0001	0.0169	X	ns	ns	ns
	Late	0.0001	0.0007	0.0214	ns	X	ns	ns
SCLC	Lim	0.0002	0.0004	<0.0001	ns	ns	X	0.0011
	Ext	<0.0001	<0.0001	ns	ns	ns	0.0011	X
**ALPHA 1-ANTITRYPSIN**								
**ANOVA Summary**			Unadjusted *p*-Value			FDR ^1^ Adjusted *p*-Value		
			<0.0001			0.0002		
**Tukey’s multiple comparison tests**								
		Control	AC		SCC		SCLC	
			Early	Late	Early	Late	Lim	Ext
Control		X	<0.0001	<0.0001	<0.0001	<0.0001	ns	<0.0001
AC	Early	<0.0001	X	<0.0001	<0.0001	<0.0001	ns	0.0034
	Late	<0.0001	<0.0001	X	ns	ns	<0.0001	ns
SCC	Early	<0.0001	<0.0001	ns	X	ns	<0.0001	ns
	Late	<0.0001	<0.0001	ns	ns	X	0.0002	ns
SCLC	Lim	ns	ns	<0.0001	<0.0001	0.0002	X	0.0003
	Ext	<0.0001	0.0034	ns	ns	ns	0.0003	X

Legend: ^1^ FDR, false discovery rate; AC, adenocarcinoma; Early, early-stage (stages 1–3a); Ext, extensive (late-stage); Late, late-stage (stages 3b–4); Lim, limited (early-stage); ns, not statistically significant at the 5% level; SCC, squamous cell carcinoma; SCLC, small cell lung cancer.

Glycosylation of carrier proteins revealed two patterns of changes between LC and control specimens (Figure 4B, Figures S9D and S10C, Table S5). The glycosylation level of haptoglobin and hemopexin was lower for control specimens across all LC groups with a stage-dependent increase in glycosylation for all subtypes of LC. Differences in glycosylation of haptoglobin between control and all LC subtypes are statistically significant, whereas for hemopexin, the glycosylation level allows differentiation of control from late-stage AC and SCC, as well as early-stage SCLC (Table S5). The opposite change in glycosylation level was observed for albumin, apolipoprotein A-I, and serotransferrin, where glycosylation was highest in the control specimens and decreased in a stage-dependent manner for all LC subtypes (Figure 4B, Figure S10D, Table 3, Table S5). These differences were statistically significant for all three proteins between control and LC subtypes, except for albumin in early stage AC (Table S5). Additionally, the glycosylation level of apolipoprotein A-I allowed for the separation of both early and late stage AC from all other subgroups (except late stage AC compared with late stage SCLC) and statistically significant differences in glycosylation of albumin were also observed for late stage SCLC and all other LC subtypes except early stage SCC (Table 3 and Table S5). In contrast, there were no statistically significant changes in the level of glycosylation in Apolipoprotein B-100 (Table S5).

Interestingly, we noticed some stage-dependent increases in glycosylation of alpha-1-antitrypsin and alpha-1-antichymotrypsin, two proteins belonging to the serpins superfamily (Figure 4C, Figures S9E and S10D, Table 3 and Table S5). However, those differences were only statistically significant for alpha-1-antitrypsin, which differentiated controls from all LC groups with the exception of early stage SCLC. Among LC groups, statistically significant differences were observed between early stage AC and both stages of SCC or late stage SCLC or AC. Moreover, statistically significant differences were also observed between early stage SCLC and late stage AC or SCLC, as well as both stages of SCC (Table 3). Some variation in glycosylation level was also observed for the remaining proteins (Figure S9F), which was expected due to the varying roles of these molecules. Since only a small number of peptides were observed for these proteins, statistical testing was not warranted.

### 3.5. Correlation between DSC Curve Summary Metrics and Overall and Progression-Free Survival of Patients

**Figure 5:**
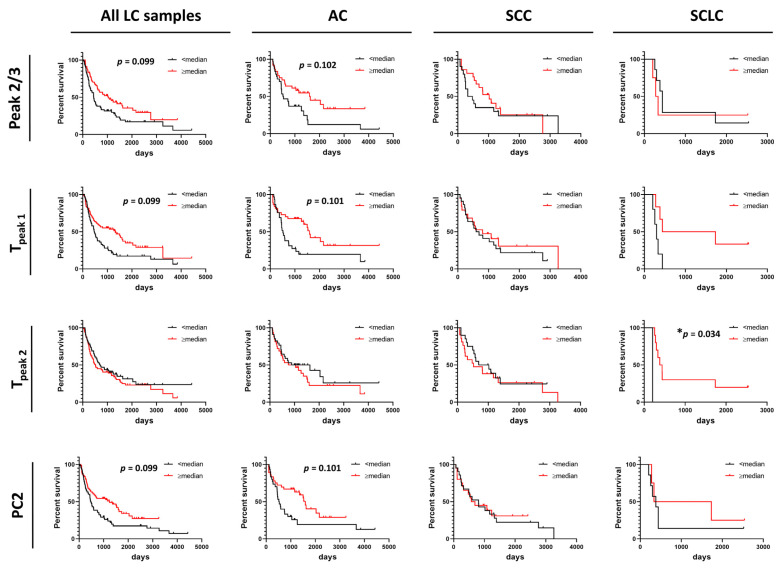
Association between overall survival of lung cancer patients and summary metrics or PCs. Analysis of survival functions for overall survival of lung cancer patients dichotomized by median values of selected summary metrics/PCs, using the Mantel–Cox test. Graphs in the first column represent data for all combined lung cancer patients (*n* = 133), columns two to four represent patients dichotomized by LC subtypes: AC LC (*n* = 66), SCC LC (*n* = 41) and SCLC (*n* = 11), respectively. *, adjusted *p*-value < 0.05.

**Figure 6:**
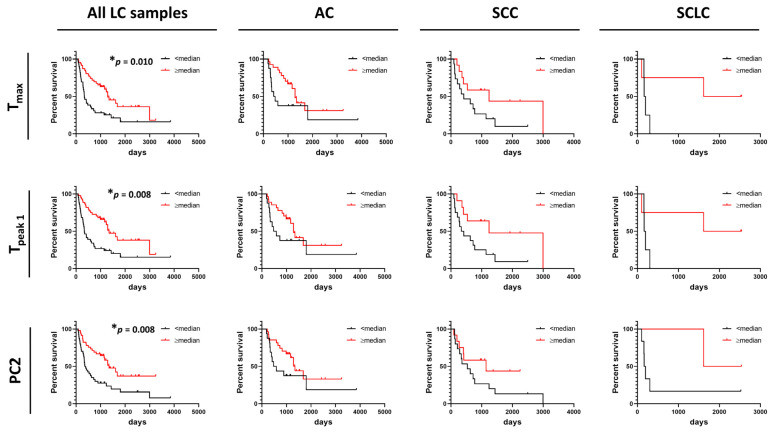
Association between progression-free survival of lung cancer patients and summary metrics or PCs. Analysis of survival functions for progression free survival of lung cancer patients dichotomized by median values of selected summary metrics/PCs, using the Mantel–Cox test. Graphs in the first column represent data for all combined lung cancer patients (*n* = 85), columns two to four represent patients dichotomized by LC subtypes: AC LC (*n* = 43), SCC LC (*n* = 27), and SCLC (*n* = 8), respectively. *, adjusted *p*-value < 0.05.

Summary metrics and/or PCs were evaluated for their use as predictors for progression-free survival (PFS) or overall survival (OS) of patients (Figure 5 and Figure 6 and Figures S11–S14). Data were dichotomized based on the value of each summary metric or PC into two groups: metric/PC values above or equal to the median value calculated for all 148 baseline LC specimens, metric/PC values below the median value calculated for all 148 baseline LC specimens. Survival functions were constructed for both PFS and OS for each of the 13 summary metrics and 6 PCs for the two groups (≥median, <median) for all LC patients combined, as well as by LC type (AC, SCC, SCLC). Detailed information about specimen data that were included in survival analysis can be found in Table S1. Additionally, we dichotomized specimens into two groups for values of T_peak 1_ and T_peak 2_ that fall within or outside the IQR (calculated for all LC specimens) to better understand the association of Peak 1 and Peak 2 positions with PFS and OS. After adjusting for multiple comparisons, no individual parameter was significant at the 5% level; however, our analysis indicated possible utility of Peak 2/3 (*p*-value = 0.014, adj. *p*-value = 0.099), T_peak 1_ (median; *p*-value = 0.008, adj. *p*-value = 0.099) and PC2 (*p*-value = 0.013, adj. *p*-value = 0.099) in predicting OS of LC patients (Figure 5) and would be of interest in future work with larger DSC data sets. Among LC subtypes, we found T_peak 2_ (based on median dichotomization) was significantly associated with OS in SCLC (*p*-value = 0.002, adj. *p*-value = 0.034; Figure 5). However, results of the survival analysis for the SCLC group should be treated with caution given the low sample size for this group, although the interesting trends can be pursued in future studies with larger patient numbers.

In contrast to OS, DSC curves were found to be useful predictors of PFS as we found that higher values of T_max_, T_peak 1_, and PC2 were significantly associated with PFS of LC patients (Figure 6). However, no association between these parameters and PFS were found for AC, SCC, and SCLC when analyzed separately. This might be explained by the low power of the study resulting from the limited number of patients for whom we had information about PFS time. Observations of the potential utility of DSC curve parameters in distinguishing patient survival would be of interest to pursue in future expanded studies.

## 4. Discussion

**Figure 7:**
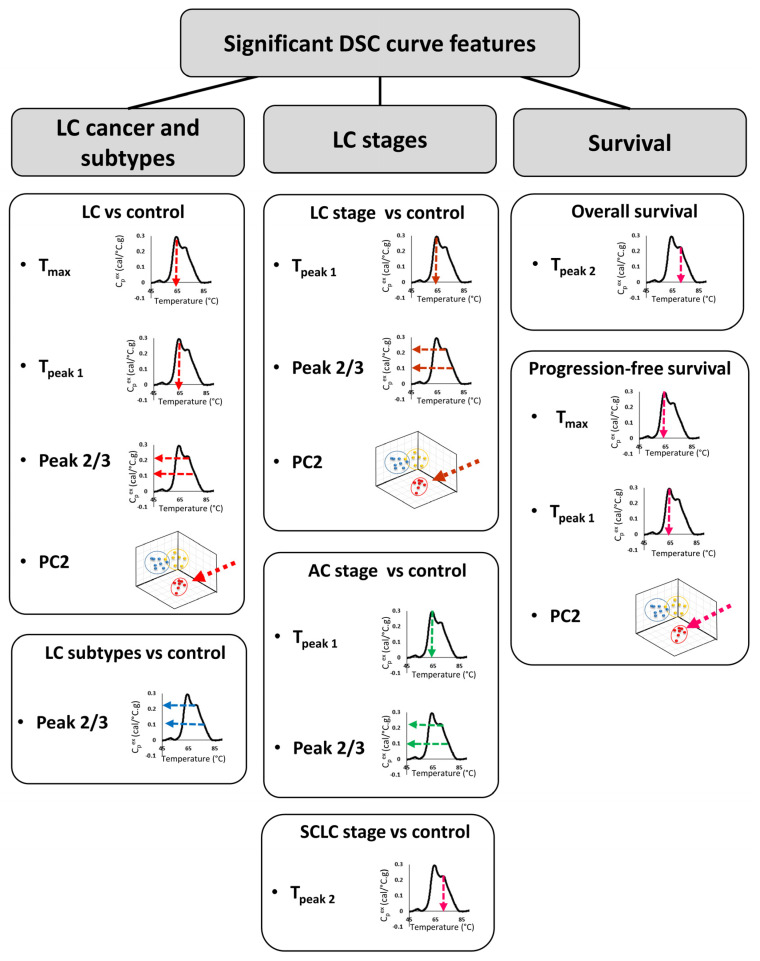
Summary of DSC curve features that were found to differentiate LC type, stage, and survival.

In the last ~15 years, DSC analysis of biofluids, including plasma, has attracted more attention as a possible method for the differentiation of healthy subjects from patients with different types of diseases, including cervical cancer, breast cancer, colorectal cancer, multiple myeloma, and brain tumors [8,9,10,11,12,13,14]. In the current study, DSC analysis of plasma specimens obtained from 148 LC patients and 51 control individuals with benign nodules revealed differences in DSC curves that could serve as potential biomarkers. We evaluated 19 parameters derived from DSC curves for the differentiation of multiple clinical groups. Although our primary consideration of differences between groups was using FDR adjusted *p*-values at the 5% significance level, given the large number of parameters investigated, we also evaluated results at less stringent type-I error rates, and presented both unadjusted and adjusted *p*-values. Several summary metrics (Peak 2/3, T_max_, T_Peak 1_) and principal components (PC2) derived from DSC curves were found to be significantly different between control and LC groups (Figure 7). When analyzed individually, ROC curves indicated that none of the DSC curve summary metrics or PCs were particularly predictive since the highest AUC value was 0.67. These results are in agreement with values of ROC curves obtained for DSC curve-derived parameters for LC patients and healthy donors published by Rodrigo et al. [24]. However, these authors expanded their approaches of DSC curve analysis to the construction of classification models and predictive values, which allowed them to increase the specificity and sensitivity of patient classification. This indicates that more complex algorithms are needed to be developed to better characterize the differences between DSC curves of plasma specimens from cancer patients and healthy subjects. It is worth pointing out that Rodrigo et al. obtained specimens for DSC analysis from a quite homogenous LC patient group that consisted mainly of Spanish Caucasian males (83%) with advanced tumors (68% stage 4 and 27% stage 3) that at the time of plasma collection were undergoing various treatments [24]. These clinical parameters could potentially affect DSC curves and could explain why the authors were able to classify patients according to the type of tumor but not by LC stage. In contrast, our analyses indicate that some of the summary metrics and PCs were statistically different between control and different subtypes of LC as well as between control and different stages (early/late), for both combined LC cases as well as within one LC subtype.

High heterogeneity in plasma/serum DSC curves within cancer types has been shown in several studies [9,25,26]. We observed similar heterogeneity in this study, which we evaluated not only by visual comparison of DSC curves but also through analysis of the distribution of summary metrics and PCs for LC groups when compared with control subjects. We found that T_max_, T_peak 1_, Peak 2/3, and PC2 obtained from DSC curves have lower values for LC patients than the control group. Moreover, in 63% of LC patients, T_Peak2_ is located outside the IQR calculated for the control group. This suggests that not only is T_Peak2_ more variant for LC patients compared to the control group, but that there is also high variability in T_Peak2_ values among LC patients. After adjusting for false discovery rate, these observations were significant at the 15–31% level and are of interest for future evaluation with the availability of additional DSC data.

In order to evaluate the use of DSC parameters as prognostic criteria, we compared OS and PFS of LC patients dichotomized based on the value of each summary metric or PC. We found that only T_peak 2_ (median) was significantly associated with OS of SCLC patients, however, among all LC patients, we observed association between OS and T_peak 1_, Peak 2/3 and PC2 at the 10–12% significance level. In contrast, in patients with glioblastoma, OS was associated with the area under the curve [27]. Interestingly, T_max_ was found to be a predictor of PFS in both LC and glioblastoma patients; however, whereas in LC higher values of T_max_ were associated with longer periods of PFS, in glioblastoma this association was reversed. Additionally, in LC patients, T_peak 1_ (median) and PC2 values were also found to be prognostic parameters for PFS. Observed differences between LC and glioblastoma could indicate that different DSC curve parameters could have prognostic value for different cancers.

Several studies suggest that DSC curves can be used to evaluate the efficacy of anti-cancer treatment. DSC curves of serum from recovering breast cancer patients were found to be useful in the prediction of tumor relapse [28], whereas a preliminary study performed on 11 metastatic LC patients found that DSC curves of serum specimens predicted the response of patients to platinum-based chemotherapy [25]. Unfortunately, since our study consisted of specimens collected from patients that were either treatment naïve or 30 days after the last dose of chemotherapy, we were unable to verify these observations in our cohort.

To have a better insight into the possible mechanism affecting the DSC curves of LC patients, we evaluated the glycosylation levels of plasma proteins. Several different PTMs have been shown to change during cancer development, aberrant protein glycosylation, differences in the level of glycoprotein, or glycan structure alteration have been widely accepted as indicators of tumor development. It has been shown that glycan structures and their compositions are differentially expressed in AC tissue compared to nonmalignant tissue from the same individuals [19,29,30]. Moreover, since patient specimens consisted not only of tumor cells but also of the surrounding stroma and other components of the intracellular environment, the authors hypothesized that the presence of these additional components might have an impact on the level and composition of observed glycoproteins [19]. In another study, SELDI-TOF MS analysis coupled with lectin-coupled ProteinChip arrays allowed for the identification of 41 glycoproteins, showing significant differences in abundance between cancer and control groups, demonstrating that changes in serum glycoprotein levels could be used as possible biomarkers for lung cancer [31]. Similarly, several studies showed alteration of N-glycosylation levels in patients with lung cancer [32,33]. Additionally, some of those changes were detectable as early as Stage 1 [18]. These data confirm that alterations in PTMs such as glycosylation allow potential differentiation of cancer from nonmalignant tissue. However, this raises a question of whether changes in PTMs caused by the tumor can be detected in the plasma/serum of affected patients. The results of several studies indicated that the presence of LC causes changes in the glycosylation profile of blood proteins [34,35,36,37,38]. These studies also lead to the identification of several possible biomarkers, including alpha-1-antichymotrypsin, apolipoprotein B, fibrinogen, hemopexin, haptoglobin, or serotransferrin [37,38,39].

The results of our study confirm that the changes in the glycosylation level of fibrinogen were highly significant, with haptoglobin, serotransferrin, and, to some degree, hemopexin, also useful in the differentiation of LC patients from control subjects. We also found highly significant differences in the glycosylation level of fibrinogen, apolipoprotein A-I, and alpha-1-antitrypsin, and to a lesser extent, albumin and immunoglobulins, between subtypes and stages of LC patients. In contrast, we have not detected any differences in the glycosylation level of alpha-1-antichymotrypsin or apolipoprotein B between different subtypes of LC patients as well as between LC and control subjects. Overall, our results show that the differences in glycosylation level of plasma proteins in LC patient specimens, when compared with controls, can partially explain differences in DSC curves. Moreover, we hypothesize that the development of classification models combining the results of DSC curve and glycosylation analyses could improve the sensitivity and specificity of the detection of LC and its subtypes.

## 5. Conclusions

In this work, we demonstrated that DSC curves are sensitive to changes in plasma composition caused by the presence of LC. Several summary metrics (T_max_, T_peak1_, and T_peak2_) and PC2 were found to be good predictors of LC patients’ OS and PFS. More importantly, some of these parameters allowed for the differentiation not only of patients with lung tumors from individuals with benign nodules but also to distinguish between subtypes and stages of LC. While no individual parameter derived from the DSC curve was sensitive and specific enough to be used as a single biomarker, we can envision that a more sophisticated classification approach incorporating several parameters of DSC curves could increase the accuracy of DSC curve-based classification, as we observed in the past for lupus patients’ data [40,41,42,43]. Although multigroup classification allowing for cancer subtype diagnosis is very challenging with high-dimensional data such as DSC curves [26], it is a crucial step for the clinical translation of DSC analysis. The accuracy of diagnosis could also be improved by combining patients’ DSC curves with results of selected plasma protein glycosylation levels, as we demonstrated that some glycosylated proteins were helpful in the differentiation of control individuals from LC patients, whereas other proteins provided differentiation by LC stage and subtype. In summary, although the utility of the DSC technique still needs to be confirmed in a clinical setting, with further optimization and development of the classification method, this technique could provide an accurate, non-invasive, radiation-free strategy for LC screening and diagnosis.

## Figures and Tables

**Table 1:** Demographic and clinical characteristics of control subjects and lung cancer patients included in the DSC analysis.

Characteristic	Control	LC Patients
*N*	51	148
Age at diagnosis: median (range)	62.0 (41–93)	58.5 (41–87)
Sex		
Female	33 (64.7%)	76 (51.4%)
Male	18 (35.3%)	72 (48.6%)
Ethnicity		
White	44 (86.3%)	116 (78.4%)
Black or African American	6 (11.8%)	31 (20.9%)
Other	1 (2.0%)	1 (0.7%)
Smoking status		
Current smokers	15 (29.4%)	64 (43.2%)
Ex-smokers	27 (52.9%)	74 (50.0%)
Never-smokers	9 (17.6%)	7 (4.7%)
Unknown	0 (0.0%)	3 (2.0%)
Lung cancer classification		
NSCLC		
AC		
Total		70 (100.0%)
Early-stage (1–3a)		36 (51.4%)
Late-stage (3b–4)		34 (48.6%)
SCC		
Total		46 (100.0%)
Early-stage (1–3a)		23 (50.0%)
Late-stage (3b–4)		23 (50.0%)
NOS		
Total		8 (100.0%)
Early-stage (1–3a)		2 (25.0%)
Late-stage (3b–4)		6 (75.0%)
Large cell carcinoma		
Total		6 (100.0%)
Early-stage (1–3a)		4 (66.7%)
Late-stage (3b–4)		2 (33.3%)
SCLC		
Total		16 (100.0%)
Early-stage (Limited)		11 (68.8%)
Late-stage (Extensive)		5 (31.3%)
Mixed type		
Total		2 (100.0%)
Early-stage		1 (50.0%)
Late-stage		1 (50.0%)

## Data Availability

DSC data are available as supplementary materials. Data files for acquired LCMS data (RAW) and sample grouping have been deposited with MassIVE (http://massive.ucsd.edu/) data repository with the Center for Computational Mass Spectrometry at the University of California, San Diegoand made public (20 October 2021) under file MSV000088001.

## Supplementary Materials

The following are available online at https://www.mdpi.com/article/10.3390/cancers13215326/s1, Figure S1. Two complete data sets collected at the beginning and end of the study showing the reproducibility of duplicate DSC curves. Figure S2. The potential utility of summary metrics and principal components to differentiate LC patients from controls. Figure S3. Smoking-related differences in DSC curves of LC and control specimens. Figure S4. Differences in DSC curves between specimens from control subjects and patients with different subtypes of LC. Figure S5. Stage-related differences in DSC curves of LC specimens. Figure S6. Stage-related differences in DSC curves of AC specimens. Figure S7. Stage-related differences in DSC curves of SCC specimens. Figure S8. Stage-related differences in DSC curves of SCLC specimens. Figure S9. “Heat maps” of glycosylation profiles of plasma proteins in control and lung cancer patient specimens. Figure S10. Glycosylation profiles of selected plasma proteins in control and lung cancer patient specimens. Figure S11. Association between overall survival of lung cancer patients and summary metrics. Figure S12. Association between overall survival of lung cancer patients and PCs. Figure S13. Association between progression-free survival of lung cancer patients and summary metrics. Figure S14. Association between progression-free survival of lung cancer patients and PCs. Table S1. Detailed patient characteristics and description of specimens included in each analysis. Table S2. DSC curve data for all patient samples included in this study. Table S3. *p*-values for the difference in summary metrics or PCs of LC and control patients across LC type, stage, smoking status, overall and progression-free survival. Table S4. Associations between summary metrics/PCs and the clinical group. Summary metrics/PCs were dichotomized based on either the median value or interquartile range (IQR) calculated for control subjects. Table S5. Summary of the *p*-values for repeated measures ANOVA and Tukey’s multiple comparisons. Tests evaluate the differences between clinical groups using mass spectrometry glycosylation data of peptides grouped according to protein function.
