# Supplementary material for: The Utility of Differential Scanning Calorimetry Curves of Blood Plasma for Diagnosis, Subtype Differentiation and Predicted Survival in Lung Cancer
Source: Cancers (Basel). 2021 Oct 23;13(21):5326. doi: 10.3390/cancers13215326 (PMC8582427; doi:10.3390/cancers13215326)
Supplement: Supplementary file 1 [file cancers-13-05326-s001.zip › cancers-1388000 -supplementary-10.26-author confirmation/cancers-1388000-Supplementary Figures FINAL 10-26-2021.pdf]

cancers

MDPI

Supplementary Materials: The Utility of Differential Scanning Calorimetry Curves of Blood Plasma for Diagnosis, Subtype Differentiation and Predicted Survival in Lung Cancer

Gabriela Schneider, Alagammai Kaliappan, Taylor Q. Nguyen, Robert Buscaglia, Guy N. Brock, Melissa Barousse Hall, Crissie DeSpirito, Daniel W. Wilkey, Michael L. Merchant, Jon B. Klein, Tanya A. Wiese, Hiram L. Rivas-Perez, Goetz H. Kloecker and Nichola C. Garbett

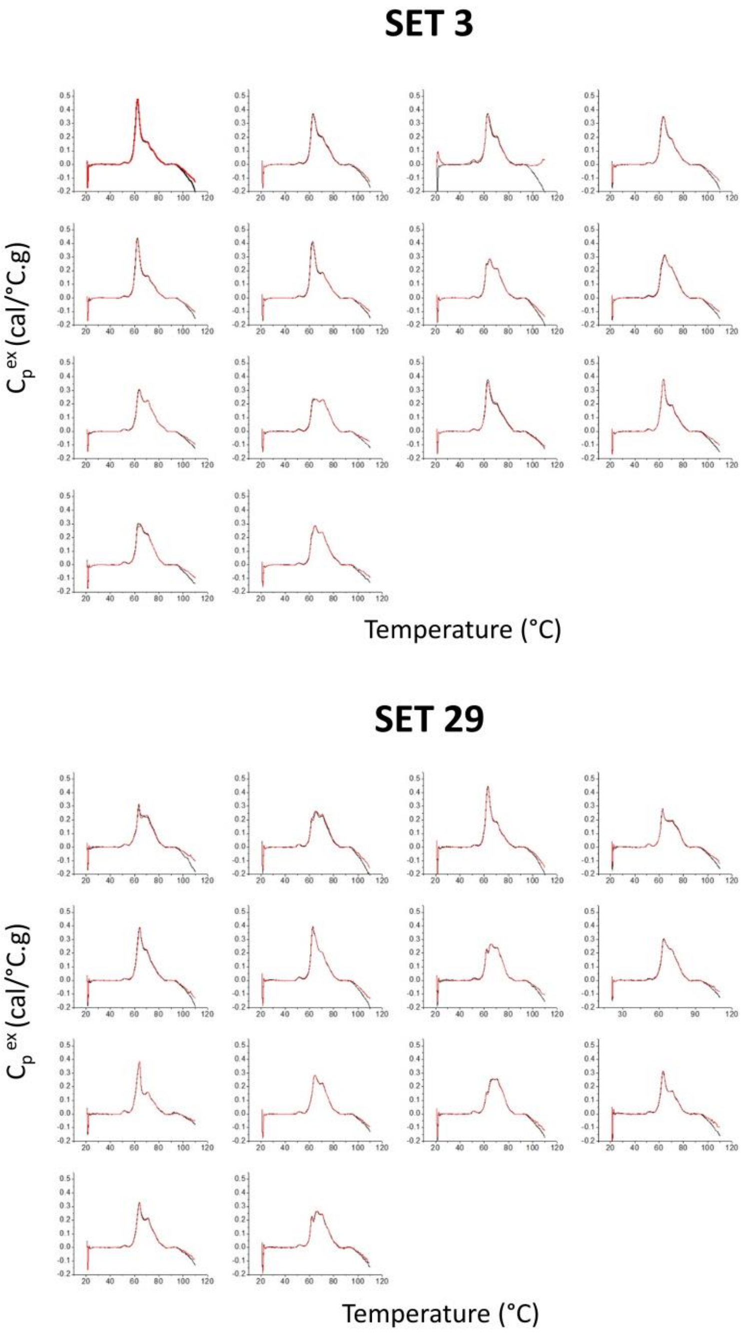

Figure S1. Two complete data sets collected at the beginning and end of the study showing the reproducibility of duplicate differential scanning calorimetry (DSC) curves.

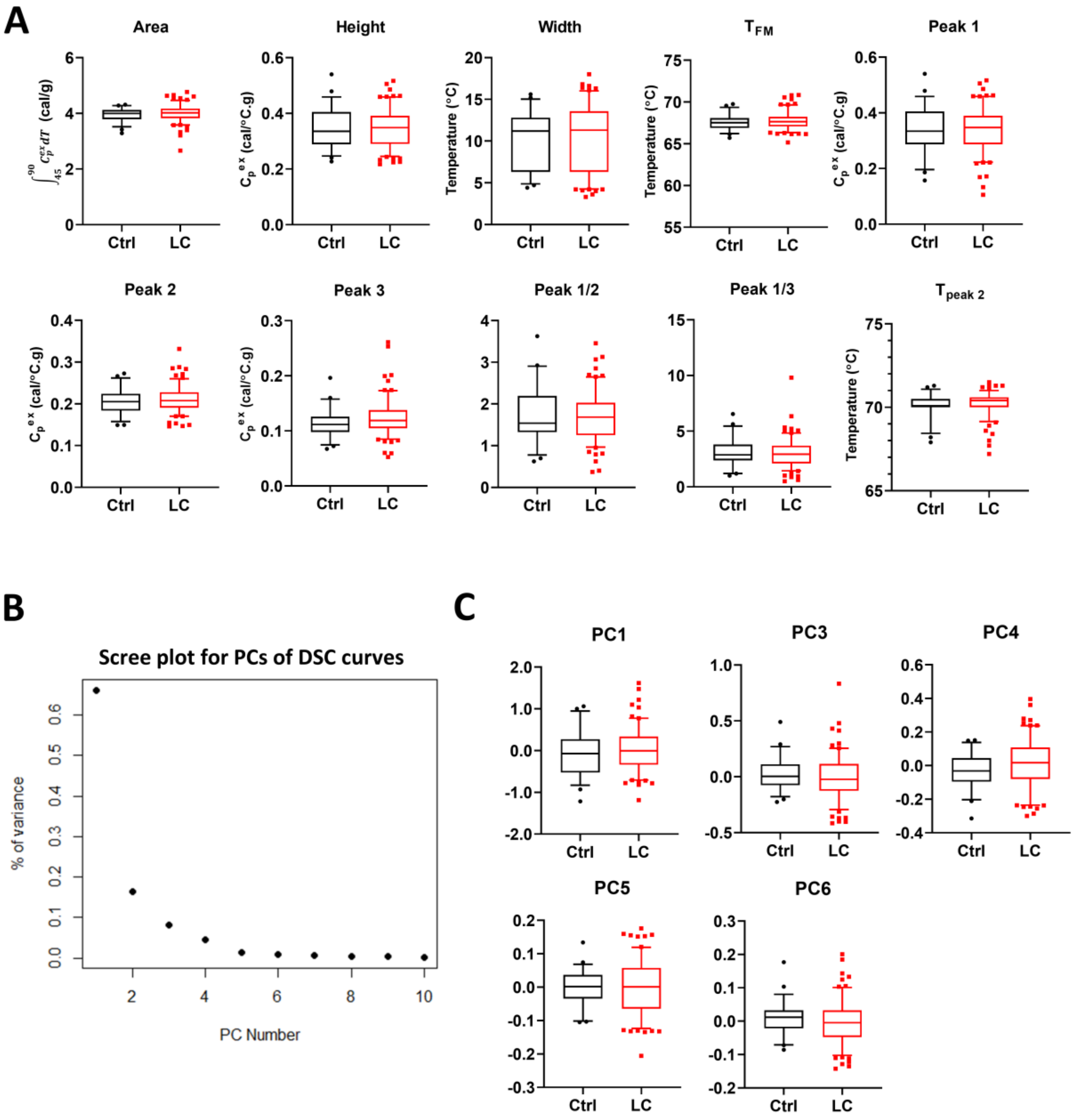

Figure S2. The potential utility of summary metrics and principal components to differentiate LC patients from controls. A. Boxplots of selected summary metrics calculated from DSC curves for LC patients and controls. B. Scree plot for principal components (PCs) of DSC curves indicating total variance in the data as explained by each PC. C. Boxplots of selected PCs calculated from DSC curves for LC patients and controls. Boxplot whiskers indicate the 5th and 95th percentiles, and points above or below the whiskers mark the values outside of this range. Ctrl, control; DSC, differential scanning calorimetry; LC, lung cancer.

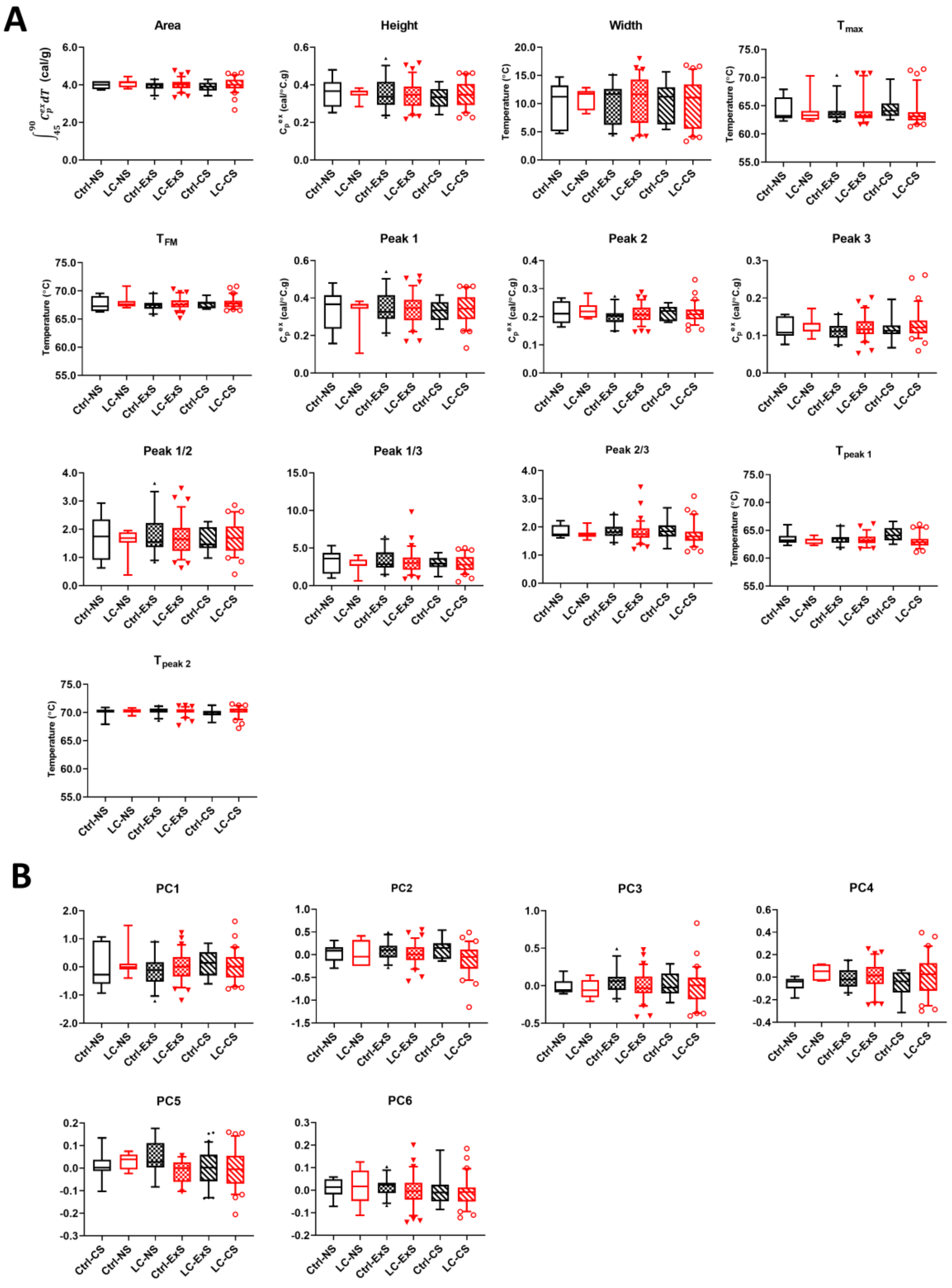

Figure S3. Smoking-related differences in DSC curves of LC and control specimens. Boxplots of selected summary metrics (**A**) or PCs (**B**) calculated from DSC curves for LC patients and control subjects dichotomized by smoking status. Boxplot whiskers indicate the 5th and 95th percentiles, and points above or below the whiskers mark the values outside of this range. Ctrl, control; CS, current smokers; DSC, differential scanning calorimetry; ExS, ex-smokers; LC, lung cancer; NS, never smokers; PC, principal component.

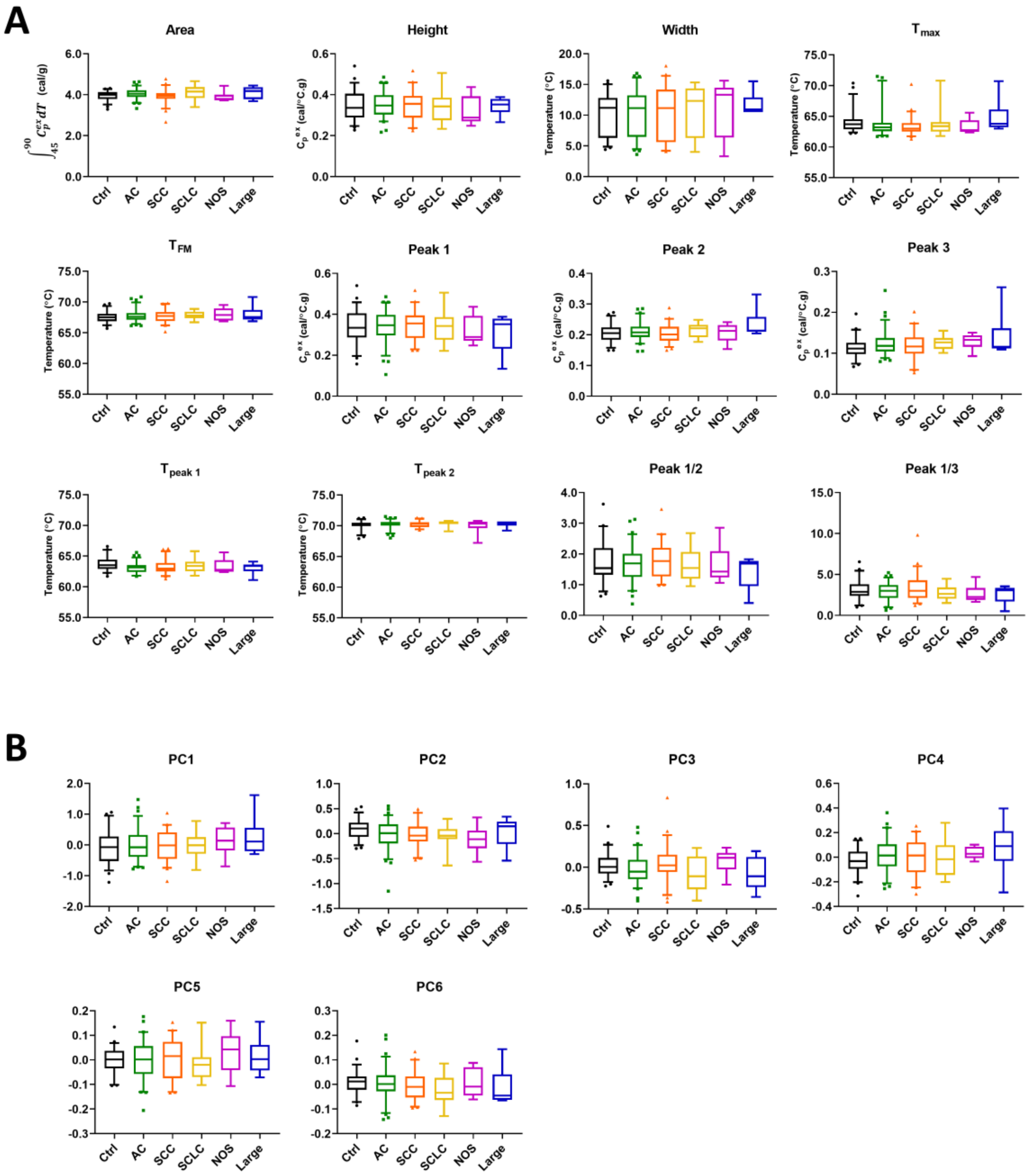

Figure S4. Differences in DSC curves between specimens from control subjects and patients with different subtypes of LC. Boxplots of summary metrics (**A**) or PCs (**B**) calculated from DSC curves for controls and different subtypes of LC patients. Boxplot whiskers indicate the 5th and 95th percentiles, and points above or below the whiskers mark the values outside of this range. AC, adenocarcinoma; Ctrl, control; DSC, differential scanning calorimetry; Large, large cell carcinoma; LC, lung cancer; NOS, not otherwise specified; PC, principal component; SCC, squamous cell carcinoma; SCLC, small cell lung cancer.

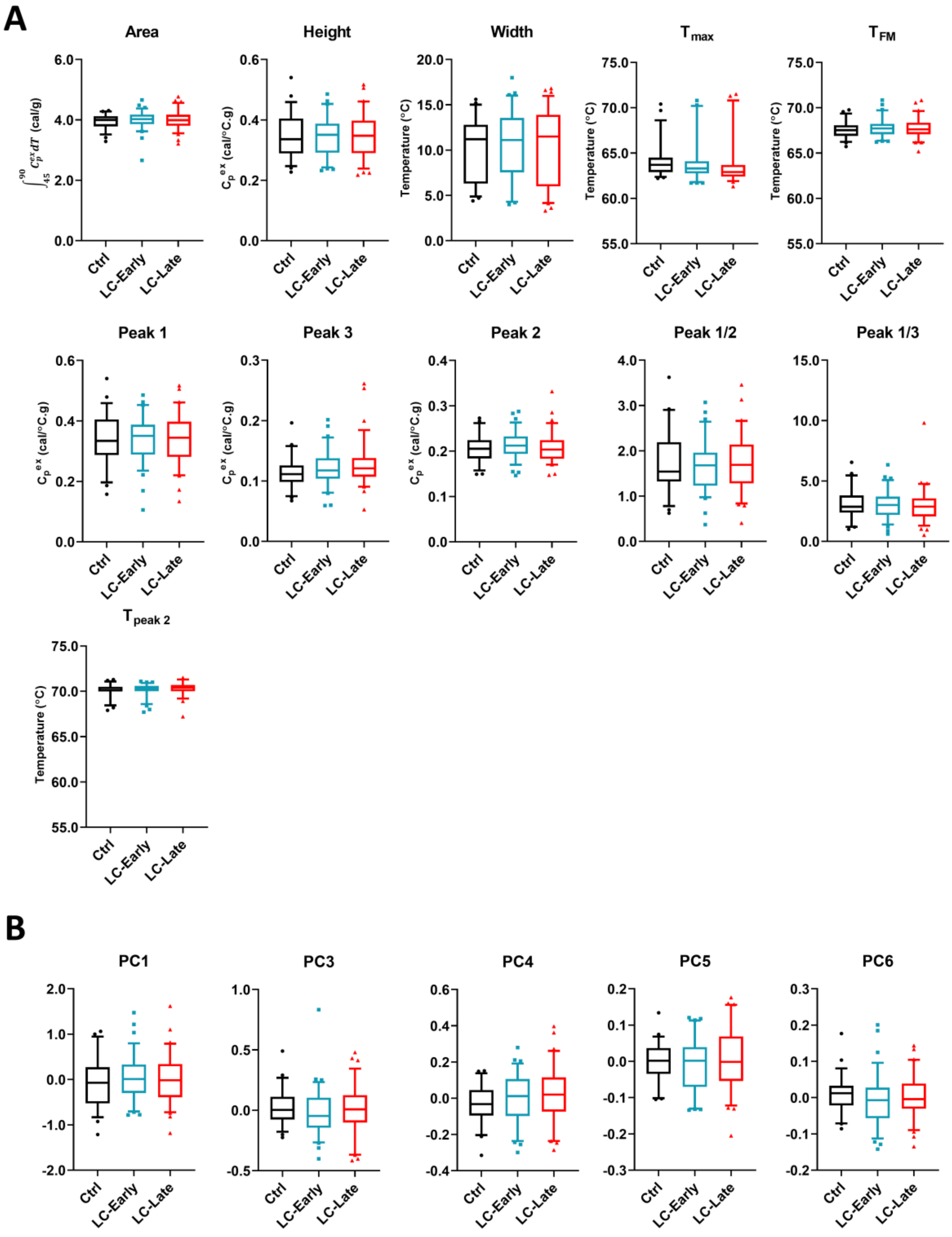

Figure S5. Stage-related differences in DSC curves of LC specimens. Boxplots of selected summary metrics (**A**) or PCs (**B**) calculated from DSC curves for controls and early (stage 1–3a) or late (stage 3b–4) stage LC patients. Boxplot whiskers indicate the 5th and 95th percentiles, and points above or below the whiskers mark the values outside of this range. Ctrl, control; DSC, differential scanning calorimetry; LC, lung cancer; PC, principal component.

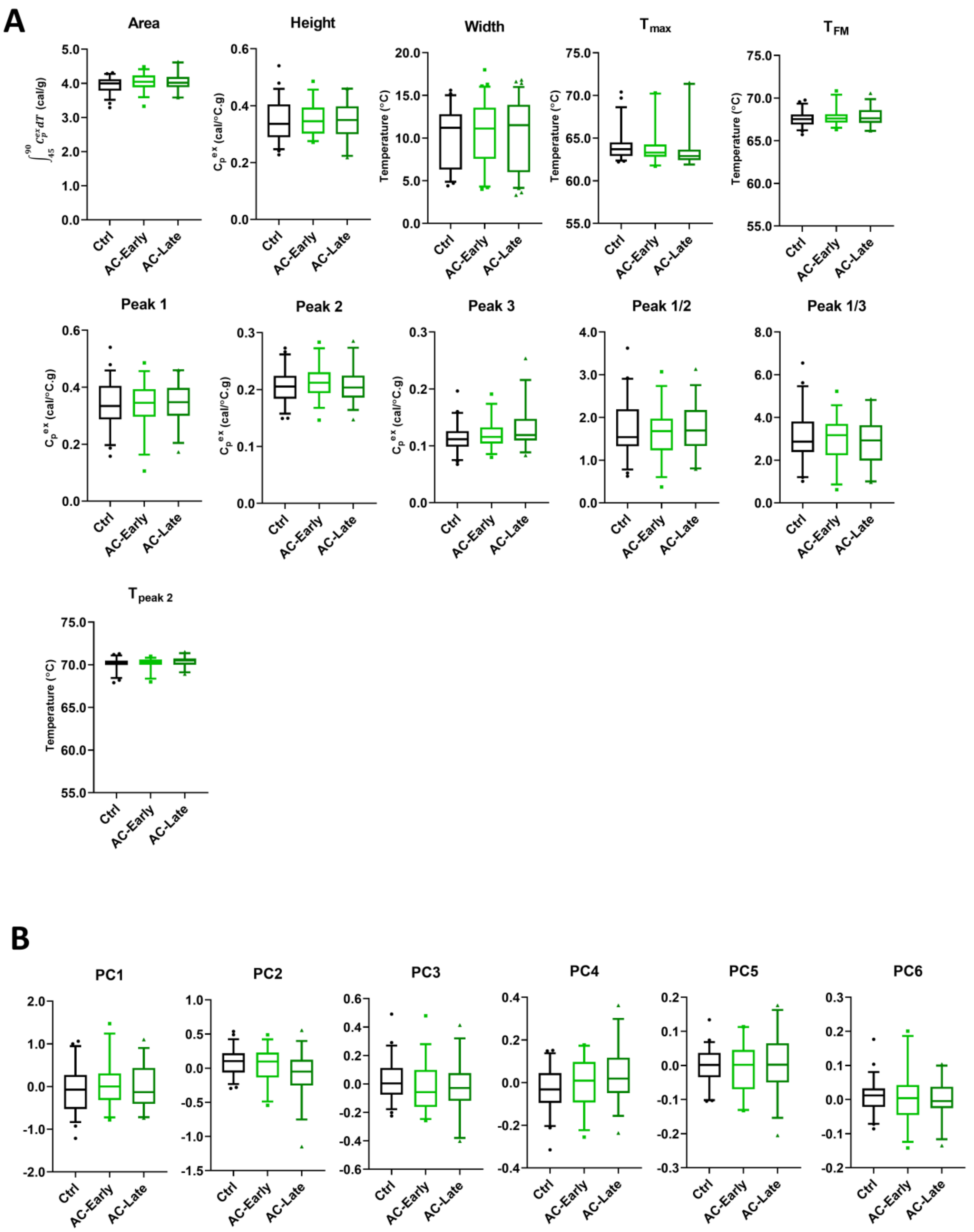

Figure S6. Stage-related differences in DSC curves of AC specimens. Boxplots of selected summary metrics (**A**) or PCs (**B**) calculated from DSC curves for patients with different stages of AC LC and control subjects. Boxplot whiskers indicate the 5th and 95th percentiles, and points above or below the whiskers mark the values outside of this range. AC, adenocarcinoma; Ctrl, control; DSC, differential scanning calorimetry; Early, early-stage (stages 1–3a); Late, late-stage (stages 3b–4); LC, lung cancer; PC, principal component.

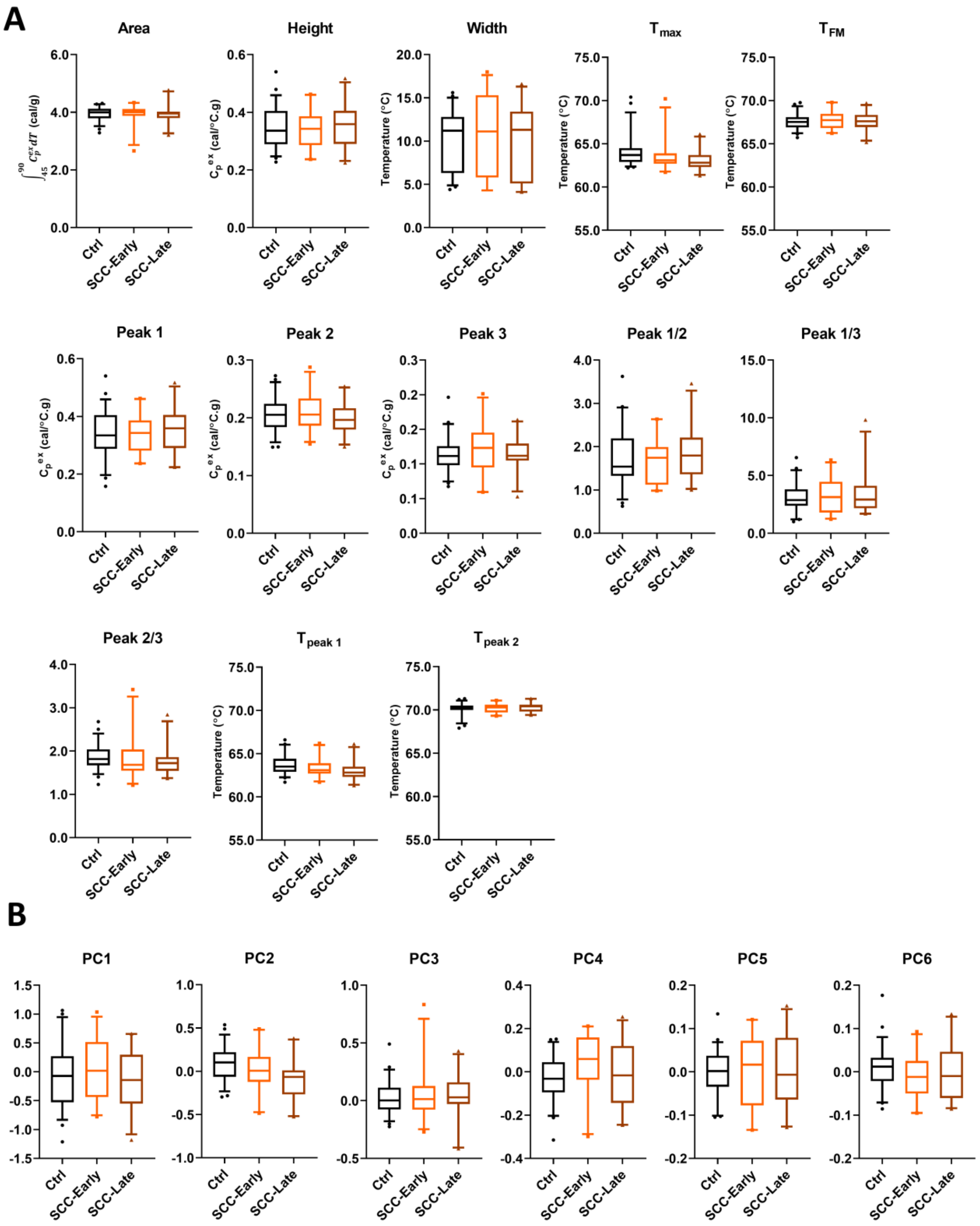

Figure S7. Stage-related differences in DSC curves of SCC specimens. Boxplots of selected summary metrics (**A**) or PCs (**B**) calculated from DSC curves for patients with different stages of SCC and control subjects. Boxplot whiskers indicate the 5th and 95th percentiles, and points above or below the whiskers mark the values outside of this range. Ctrl, control; DSC, differential scanning calorimetry; Early, early-stage (stages 1–3a); Late, late-stage (stages 3b–4); PC, principal component; SCC, squamous cell carcinoma.

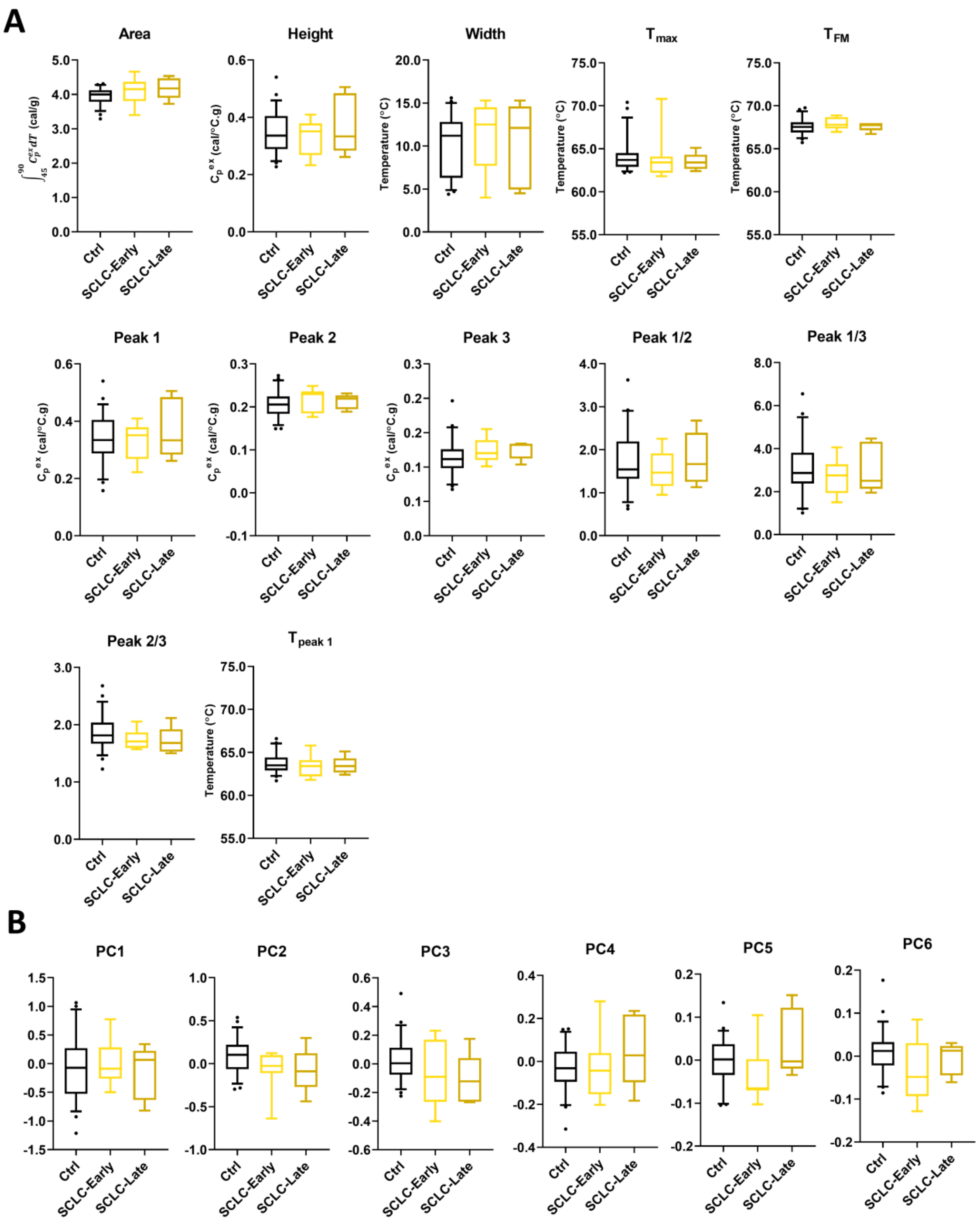

Figure S8. Stage-related differences in DSC curves of SCLC specimens. Boxplots of selected summary metrics (**A**) or PCs (**B**) calculated from DSC curves for patients with different stages of SCLC and control subjects. Boxplot whiskers indicate the 5th and 95th percentiles, and points above or below the whiskers mark the values outside of this range. Ctrl, control; DSC, differential scanning calorimetry; Early, early-stage (stages 1–3a); Late, late-stage (stages 3b–4); PC, principal component; SCLC, small cell lung cancer.

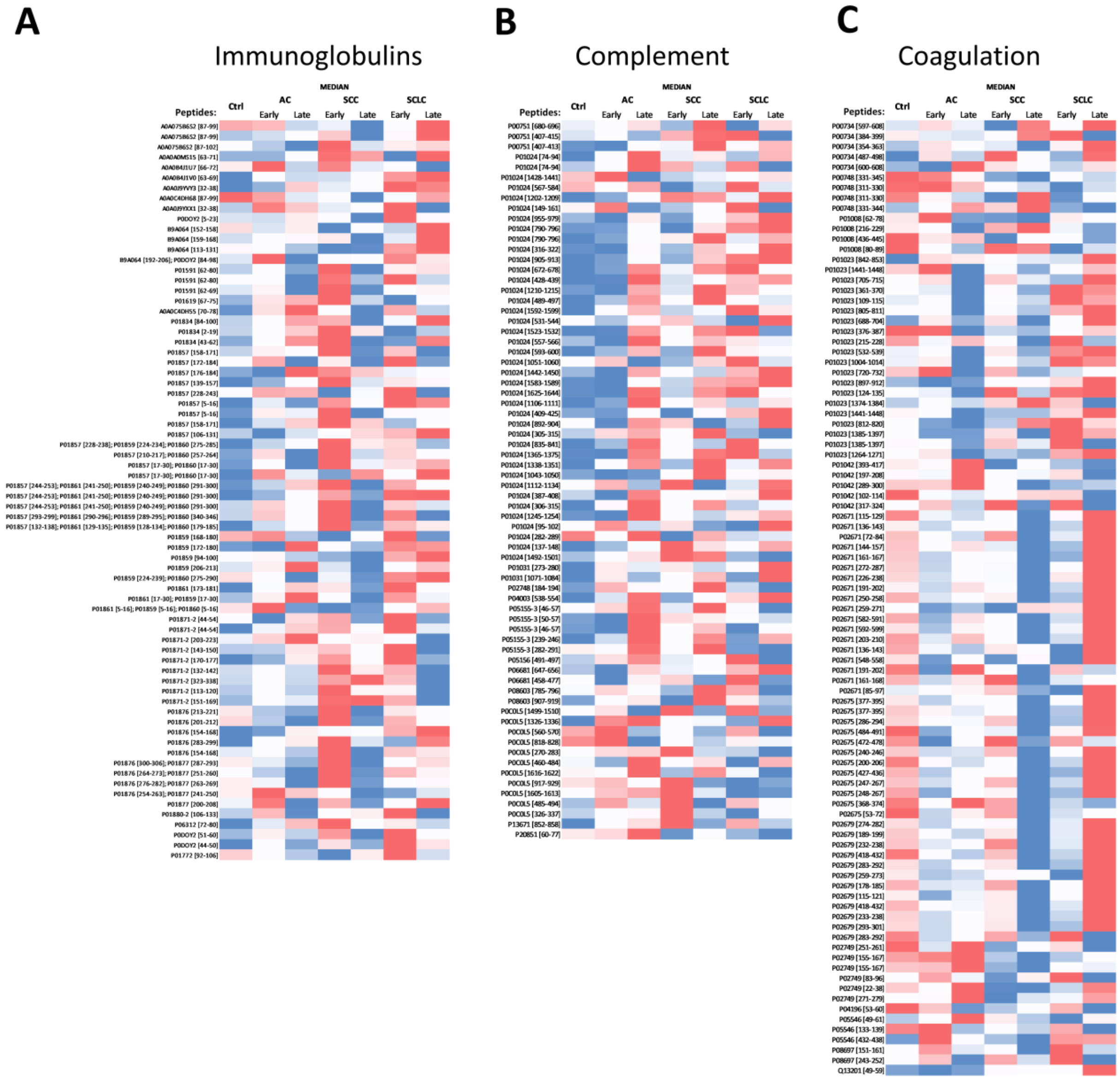

Figure S9. "Heat maps" of glycosylation profiles of plasma proteins in control and lung cancer patient specimens. "Heat maps" representing glycosylation of selected peptides that correspond to proteins involved in immune response (**A** and **B**), coagulation (**C**), transportation (**D**), the serpins family (**E**), and other selected proteins (**F**). AC, adenocarcinoma; Ctrl, control; Early, early-stage (stages 1–3a); Late, late-stage (stages 3b–4); SCC, squamous cell carcinoma; SCLC, small cell lung cancer.

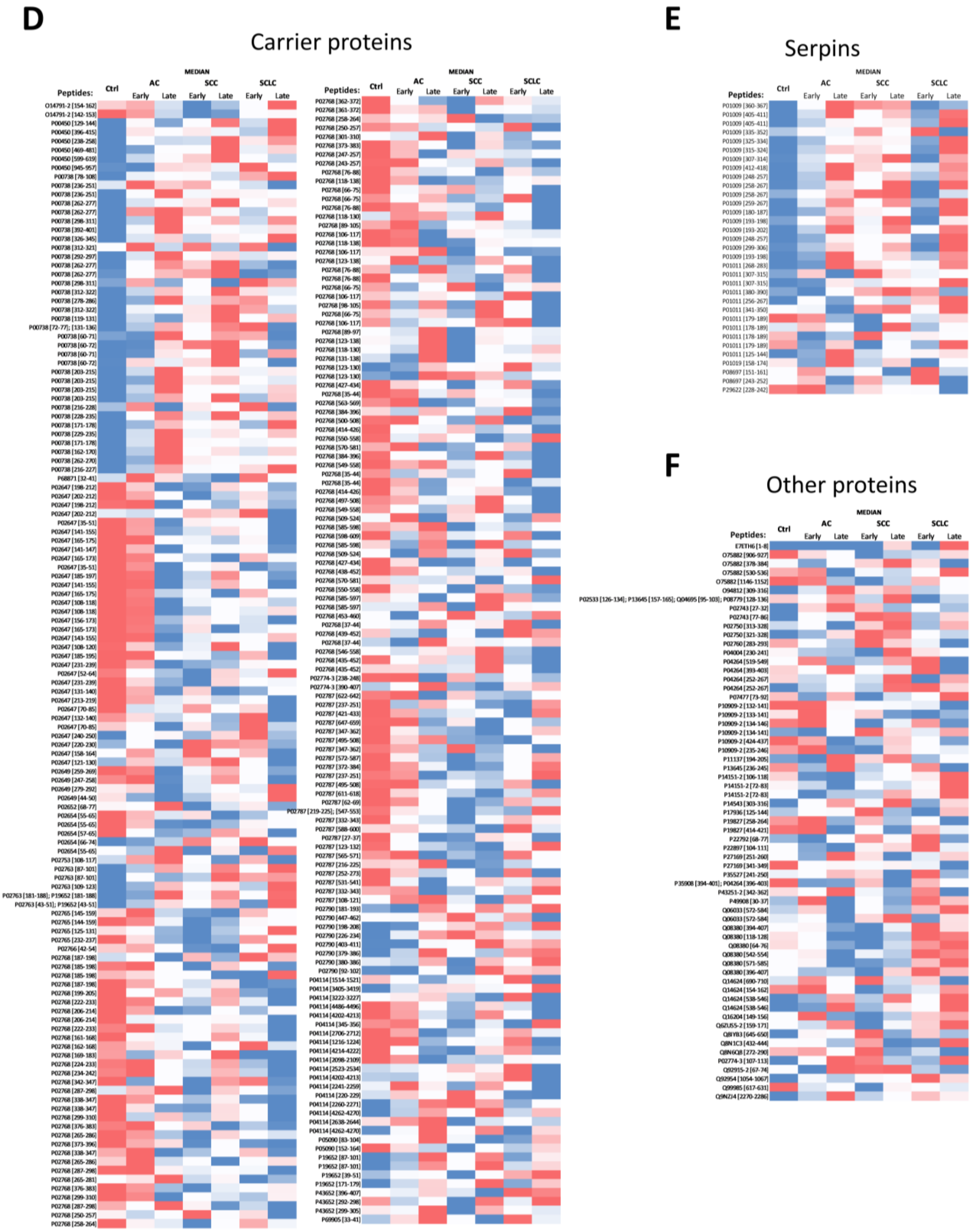

Figure S9. – continued.

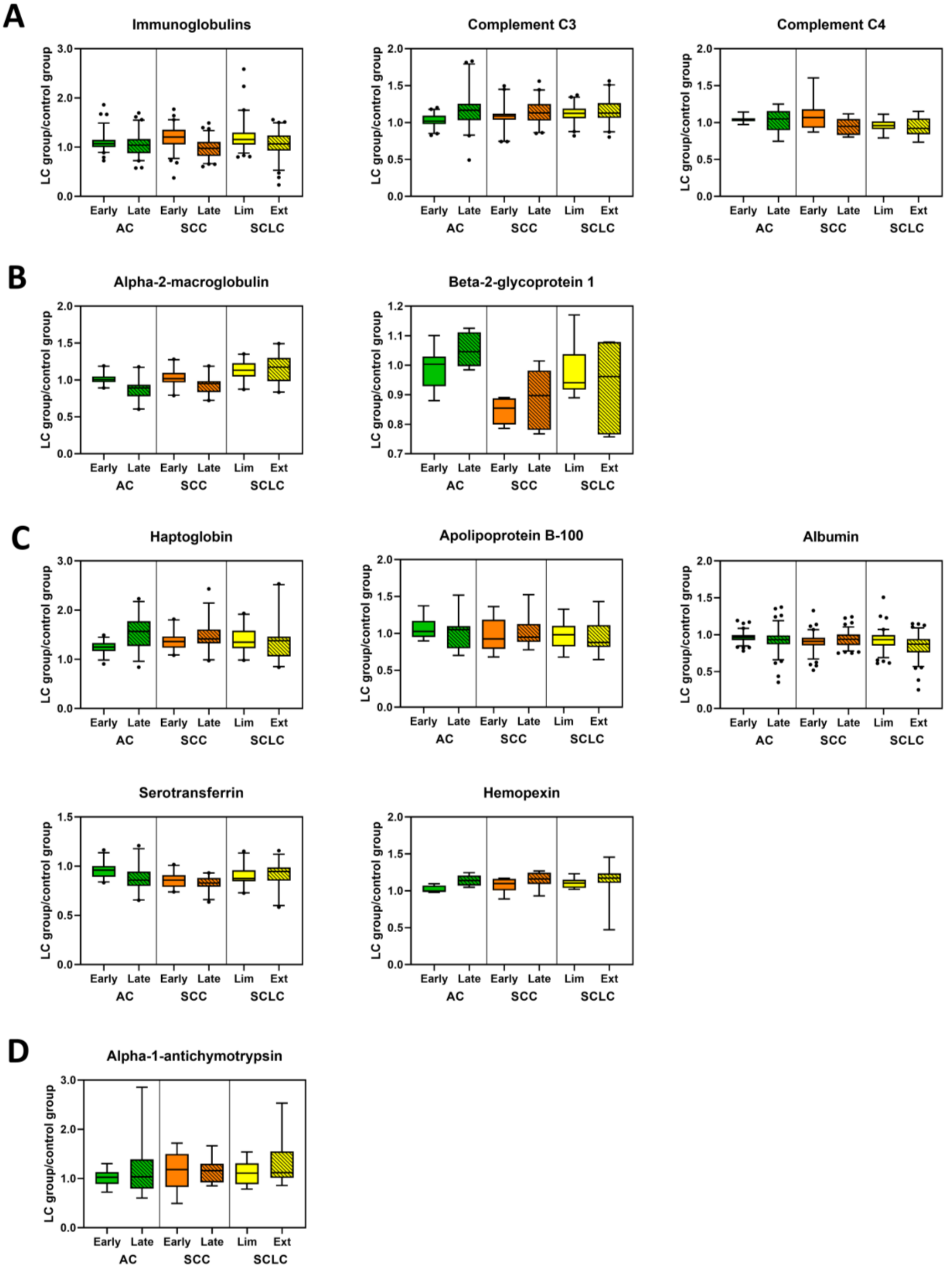

Figure S10. Glycosylation profiles of selected plasma proteins in control and lung cancer patient specimens. Examples of glycosylation levels of selected proteins involved in immune response (**A**), coagulation (**B**), transportation (**C**) or belonging to the serpins family (**D**) in patients with different stages of AC, SCC and SCLC. Data are presented normalized to control values, (LC group)/ (Control group). Boxplot whiskers indicate the 5th and 95th percentiles, and points above or below the whiskers mark the values outside of this range. AC, adenocarcinoma; Early, early-stage (stages 1–3a); Ext, extensive (late-stage); Late, late-stage (stages 3b–4); LC, lung cancer; Lim, limited (early-stage); SCC, squamous cell carcinoma; SCLC, small cell lung cancer.

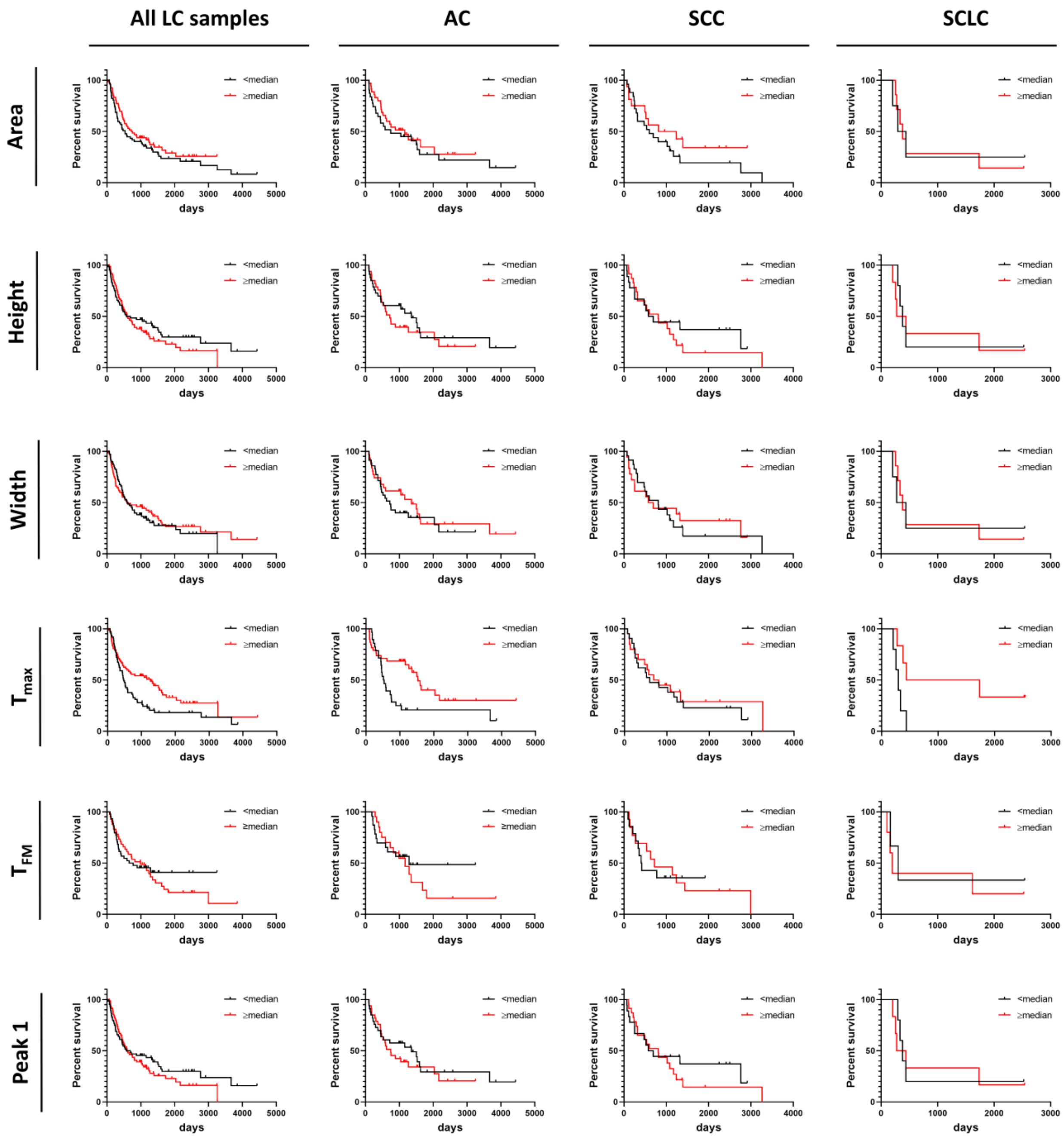

Figure S11. Association between overall survival of lung cancer patients and summary metrics. Survival functions for overall survival of lung cancer patients dichotomized by median values of selected summary metrics or IQR for $T_{peak\ 1}$ using the Mantel-Cox test. Graphs in the first column represent data for all combined lung cancer (LC) patients (*n*=133), columns 2-4 represent patients dichotomized by LC subtypes: adenocarcinoma (AC; *n*=66), squamous cell carcinoma (SCC; *n*=41) and small cell lung cancer (SCLC; *n*=11), respectively. IQR, interquartile range [Q1, Q3].

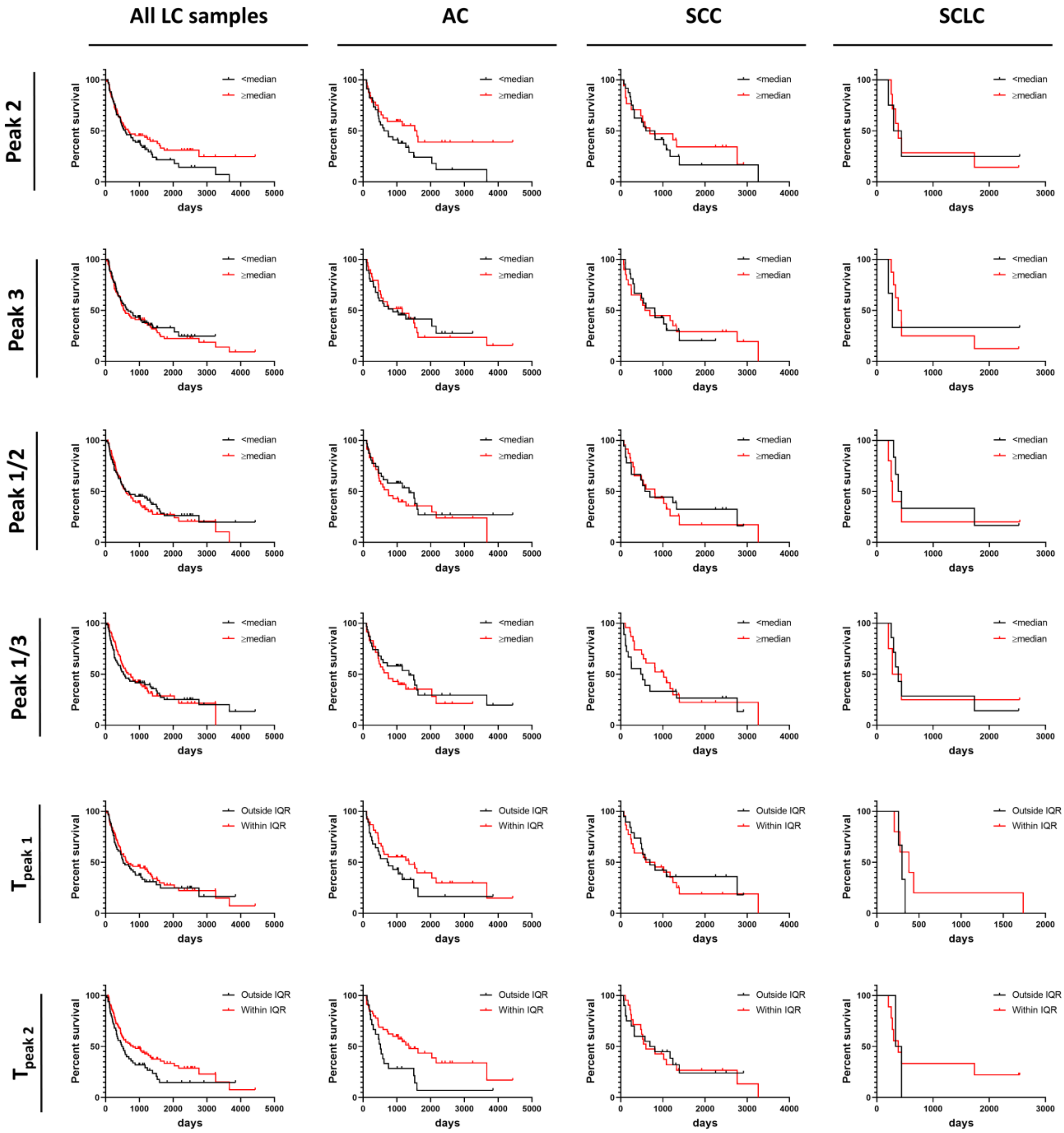

Figure S11. – continued.

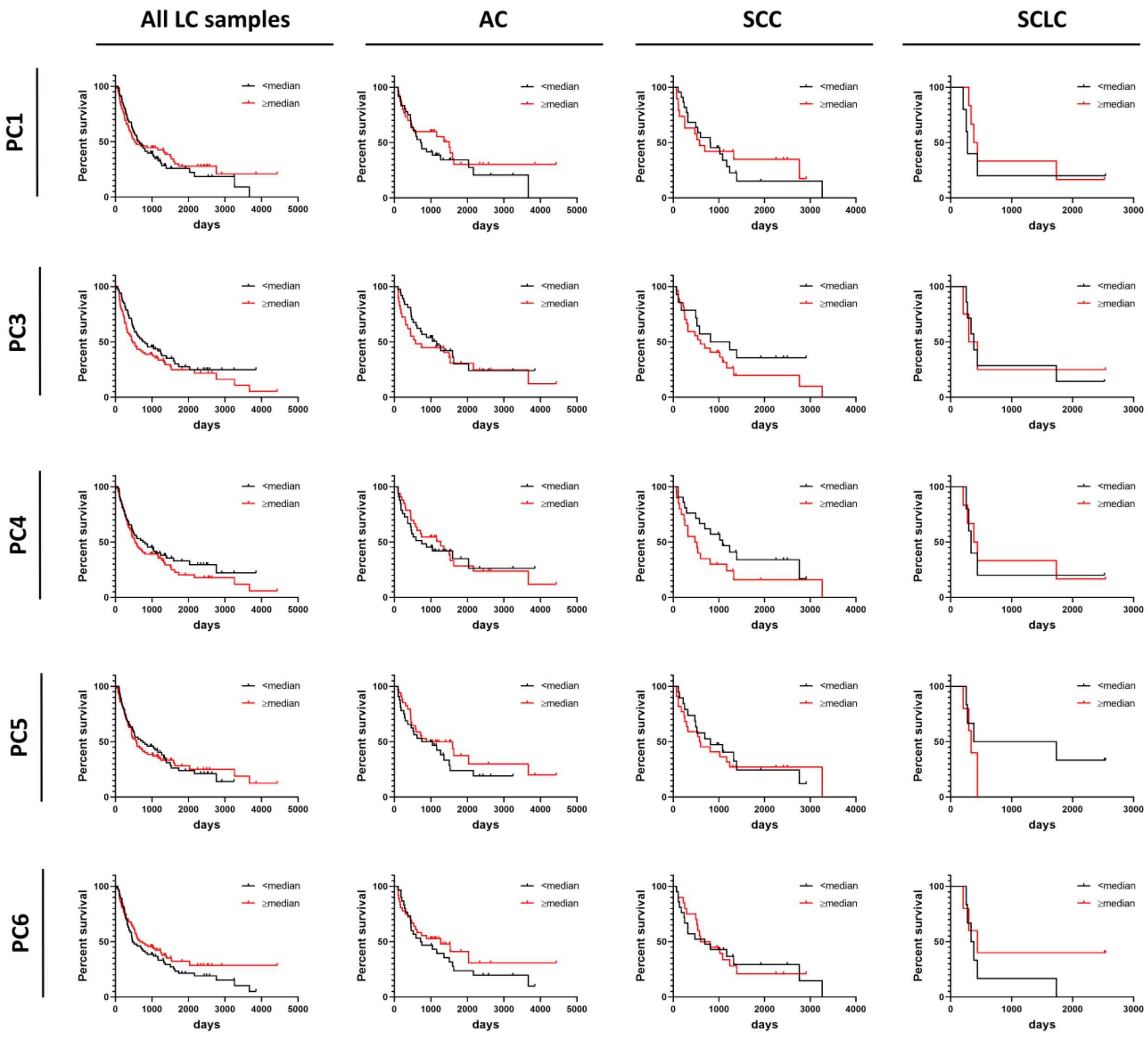

Figure S12. Association between overall survival of lung cancer patients and PCs. Survival functions for overall survival of lung cancer patients dichotomized by median values of selected PCs using the Mantel-Cox test. Graphs in the first column represent data for all combined lung cancer (LC) patients (*n*=133), columns 2-4 represent patients dichotomized by LC subtypes: adenocarcinoma (AC; *n*=66), squamous cell carcinoma (SCC; *n*=41) and small cell lung cancer (SCLC; *n*=11), respectively. PC, principal component.

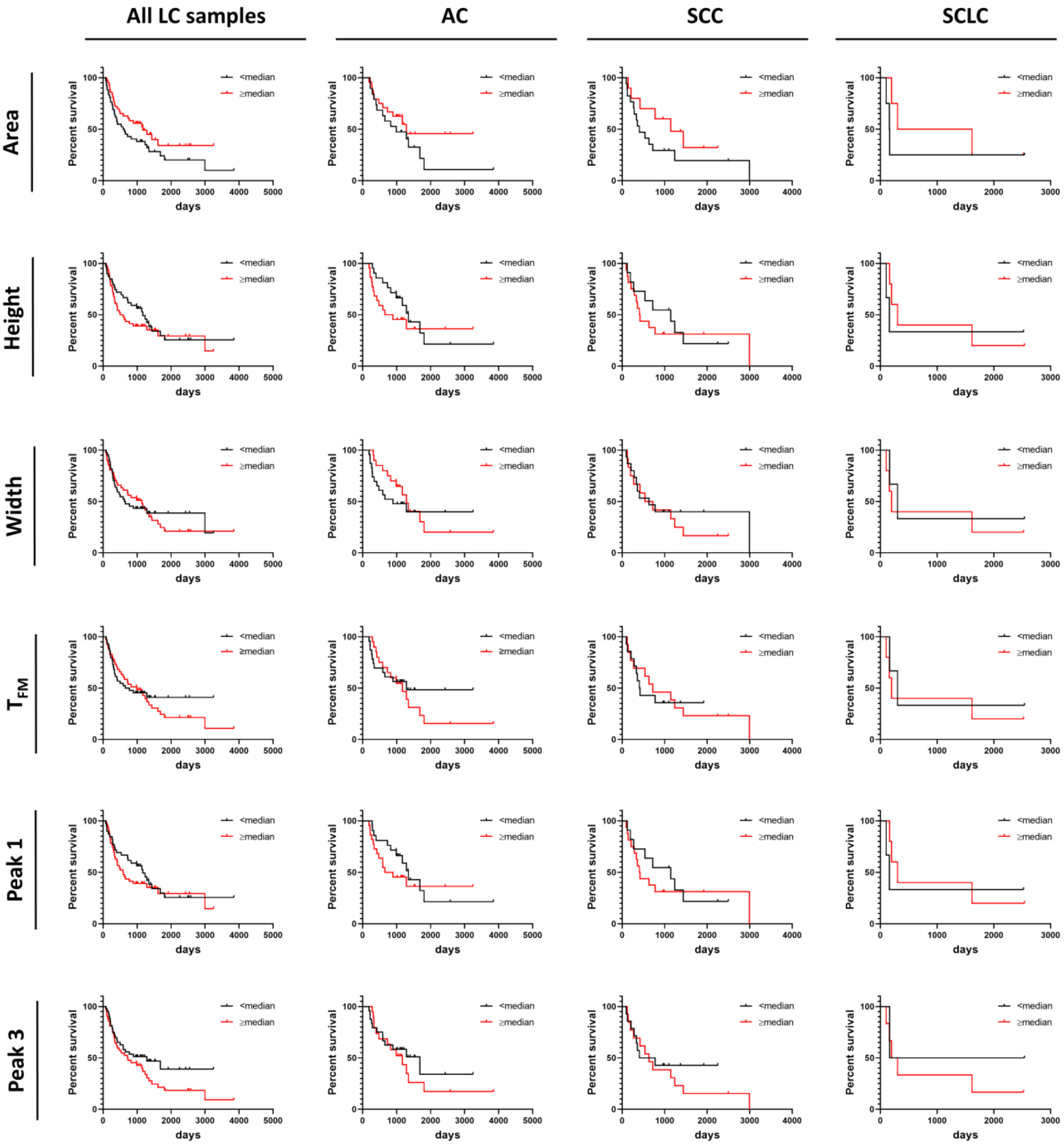

Figure S13. Association between progression-free survival of lung cancer patients and summary metrics. Survival functions for progression free survival of lung cancer patients dichotomized by median values of selected summary metrics or IQR for $T_{peak\ 1}$ using the Mantel-Cox test. Graphs in the first column represent data for all combined lung cancer (LC) patients (*n*=85), columns 2-4 represent patients dichotomized by LC subtypes: adenocarcinoma (AC; *n*=43), squamous cell carcinoma (SCC; *n*=27) and small cell lung cancer (SCLC; *n*=8), respectively. IQR, interquartile range [Q1, Q3].

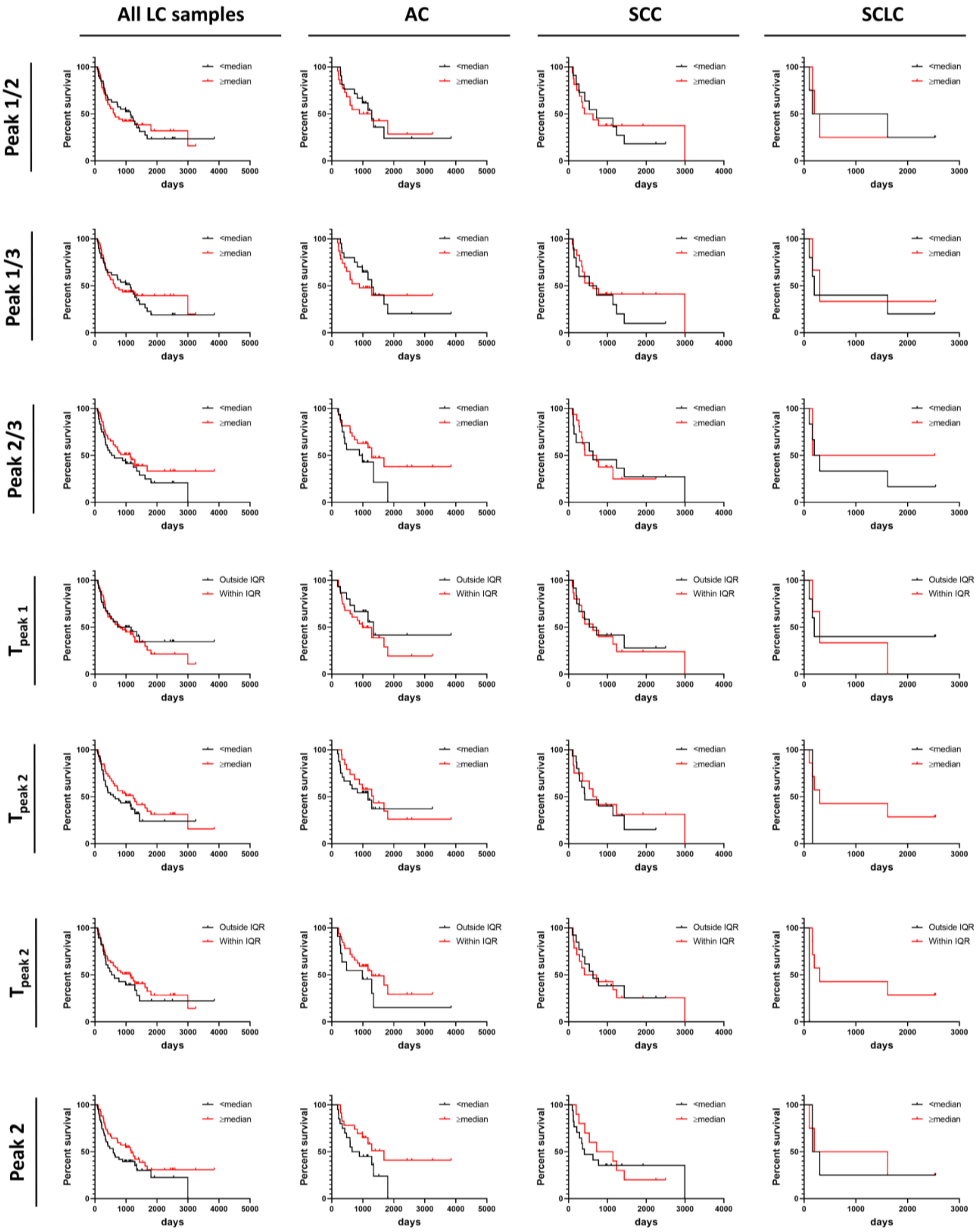

Figure S13. – continued.

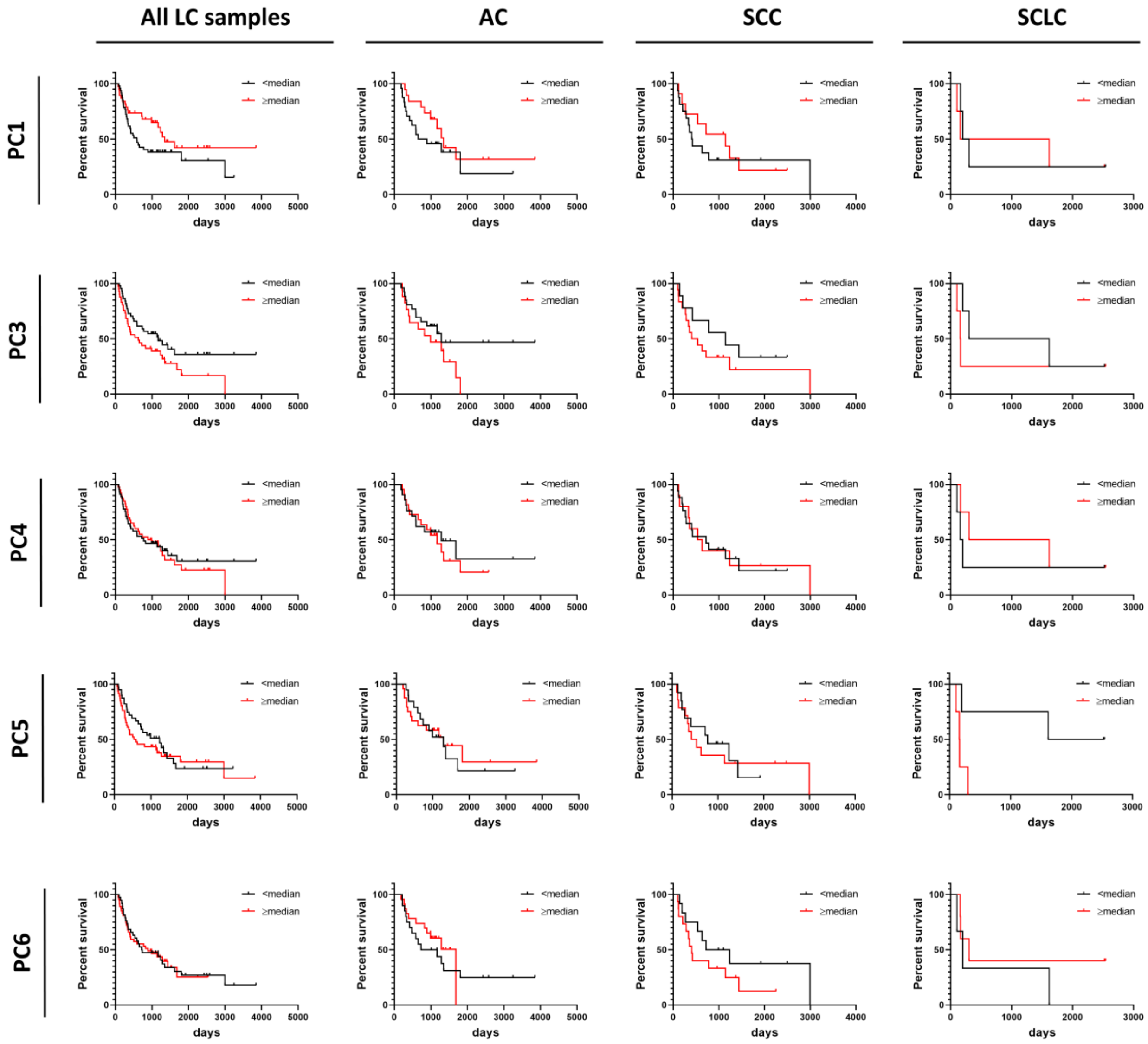

Figure S14. Association between progression-free survival of lung cancer patients and PCs. Survival functions for progression free survival of lung cancer patients dichotomized by median values of selected PCs using the Mantel-Cox test. Graphs in the first column represent data for all combined lung cancer (LC) patients (*n*=85), columns 2-4 represent patients dichotomized by LC subtypes: adenocarcinoma (AC; *n*=43), squamous cell carcinoma (SCC; *n*=27) and small cell lung cancer (SCLC; *n*=8), respectively. PC, principal component.
