# Supplementary material for: The Utility of Differential Scanning Calorimetry Curves of Blood Plasma for Diagnosis, Subtype Differentiation and Predicted Survival in Lung Cancer
Source: Cancers (Basel). 2021 Oct 23;13(21):5326. doi: 10.3390/cancers13215326 (PMC8582427; doi:10.3390/cancers13215326)
Supplement: Supplementary file 1 [file cancers-13-05326-s001.zip › cancers-1388000 -supplementary-10.26-author confirmation/cancers-1388000-Supplementary Tables.pdf]

Supplementary Materials: The Utility of Differential Scanning Calorimetry Curves of Blood Plasma for Diagnosis, Subtype Differentiation and Predicted Survival in Lung Cancer

Gabriela Schneider, Alagammai Kaliappan, Taylor Q. Nguyen, Robert Buscaglia, Guy N. Brock, Melissa Barousse Hall, Crissie DeSpirito, Daniel W. Wilkey, Michael L. Merchant, Jon B. Klein, Tanya A. Wiese, Hiram L. Rivas-Perez, Goetz H. Kloecker and Nichola C. Garbett

Table S1. Detailed patient characteristics and description of specimens included in each analysis.

Patient ID	Age	Sex	Ethnicity	Smoking Status	Diagnosis	Stage	Included in The Analysis			
							DSC	Gly	OS	PFS
C1	47	Female	White	Never smoked	Control		Yes	No	No	No
C2	41	Female	White	Ex-smoker	Control		Yes	Yes	No	No
C3	68	Female	White	Ex-smoker	Control		Yes	Yes	No	No
C4	56	Male	White	Current smoker	Control		Yes	No	No	No
C5	49	Male	White	Never smoked	Control		Yes	No	No	No
C6	59	Female	White	Ex-smoker	Control		Yes	Yes	No	No
C7	61	Female	White	Current smoker	Control		Yes	Yes	No	No
C8	66	Female	White	Current smoker	Control		Yes	Yes	No	No
C9	46	Male	Black or African American	Ex-smoker	Control		Yes	Yes	No	No
C10	48	Female	White	Current smoker	Control		Yes	Yes	No	No
C11	60	Male	White	Current smoker	Control		Yes	Yes	No	No
C12	54	Male	White	Ex-smoker	Control		Yes	Yes	No	No
C13	62	Female	White	Ex-smoker	Control		Yes	Yes	No	No
C14	63	Female	Black or African American	Ex-smoker	Control		Yes	Yes	No	No
C15	41	Female	White	Current smoker	Control		Yes	Yes	No	No
C16	75	Female	White	Ex-smoker	Control		Yes	Yes	No	No
C17	63	Female	White	Current smoker	Control		Yes	Yes	No	No
C18	62	Male	White	Ex-smoker	Control		Yes	Yes	No	No
C19	61	Female	Black or African American	Ex-smoker	Control		Yes	Yes	No	No
C20	65	Female	White	Current smoker	Control		Yes	Yes	No	No
C21	60	Male	White	Ex-smoker	Control		Yes	Yes	No	No

C22	93	Female	White	Ex-smoker	Control		Yes	Yes	No	No
C23	70	Female	White	Never smoked	Control		Yes	No	No	No
C24	80	Female	White	Current smoker	Control		Yes	Yes	No	No
C25	55	Male	White	Ex-smoker	Control		Yes	Yes	No	No
C26	55	Male	White	Never smoked	Control		Yes	No	No	No
C27	67	Male	White	Ex-smoker	Control		Yes	Yes	No	No
C28	77	Female	White	Ex-smoker	Control		Yes	Yes	No	No
C29	64	Male	White	Ex-smoker	Control		Yes	Yes	No	No
C30	80	Female	White	Never smoked	Control		Yes	No	No	No
C31	73	Female	White	Current smoker	Control		Yes	Yes	No	No
C32	86	Female	White	Ex-smoker	Control		Yes	Yes	No	No
C33	66	Male	Black or African American	Ex-smoker	Control		Yes	Yes	No	No
C34	59	Female	White	Ex-smoker	Control		Yes	Yes	No	No
C35	52	Female	White	Ex-smoker	Control		Yes	Yes	No	No
C36	65	Male	White	Current smoker	Control		Yes	Yes	No	No
C37	68	Male	White	Ex-smoker	Control		Yes	Yes	No	No
C38	58	Female	White	Ex-smoker	Control		Yes	Yes	No	No
C39	62	Female	Black or African American	Current smoker	Control		Yes	Yes	No	No
C40	54	Female	White	Ex-smoker	Control		Yes	Yes	No	No
C41	56	Male	White	Ex-smoker	Control		Yes	Yes	No	No
C42	85	Male	Pakistani	Never smoked	Control		Yes	No	No	No
C43	71	Female	White	Never smoked	Control		Yes	Yes	No	No
C44	68	Female	White	Ex-smoker	Control		Yes	Yes	No	No
C45	45	Female	White	Current smoker	Control		Yes	Yes	No	No
C46	54	Female	Black or African American	Never smoked	Control		Yes	No	No	No
C47	55	Male	White	Current smoker	Control		Yes	Yes	No	No
C48	59	Female	White	Ex-smoker	Control		Yes	Yes	No	No
C49	67	Female	White	Ex-smoker	Control		Yes	No	No	No
C50	70	Female	White	Never smoked	Control		Yes	No	No	No
C51	42	Male	White	Current Smoker	Control		Yes	No	No	No
L1	56	Female	Black or African American	Ex-smoker	AC	Late	Yes	No	298	No

L2	59	Female	White	Unknown	AC	Early	Yes	No	1538*	1538*
L3	72	Male	White	Current Smoker	AC	Early	Yes	Yes	1518*	1518*
L4	71	Female	White	Ex-smoker	AC	Early	Yes	Yes	1403*	1403*
L5	79	Male	White	Ex-smoker	SCC	Early	Yes	Yes	1372*	1372*
L6	57	Female	White	Ex-smoker	AC	Early	Yes	No	1337*	1337*
L7	64	Female	White	Ex-smoker	SCC	Early	Yes	No	574	No
L8	58	Female	White	Current Smoker	SCC	Late	Yes	No	2913*	1436
L9	55	Female	White	Ex-smoker	SCLC	Early	Yes	Yes	No	No
L10	70	Female	Black or African American	Ex-smoker	SCC	Early	Yes	No	2415*	1143
L11	59	Female	Black or African American	Ex-smoker	AC	Early	Yes	Yes	1160	736
L12	64	Female	Black or African American	Ex-smoker	SCC	Early	Yes	Yes	882	No
L13	87	Female	White	Never smoked	AC	Late	Yes	No	No	Yes
L14	76	Female	White	Current Smoker	AC	Early	Yes	No	1216*	1216*
L15	64	Male	White	Current Smoker	AC	Early	Yes	Yes	621	268
L16	80	Male	White	Ex-smoker	AC	Early	Yes	Yes	105	No
L17	48	Male	White	Current Smoker	Large	Late	Yes	No	38	No
L18	67	Male	White	Ex-smoker	SCC	Early	Yes	No	1390	775
L19	66	Male	White	Ex-smoker	Mix	Late	Yes	No	1222*	1222*
L20	53	Female	White	Current Smoker	Large	Late	Yes	No	164	111
L21	77	Female	White	Ex-smoker	SCC	Late	Yes	No	1239	419
L22	73	Male	White	Never smoked	NOS	Late	Yes	No	351	No
L23	48	Male	White	Current Smoker	SCC	Late	Yes	No	812	No
L24	57	Male	White	Current Smoker	SCC	Late	Yes	Yes	110	94
L25	74	Male	Black or African American	Ex-smoker	SCC	Early	Yes	No	476	No
L26	77	Male	White	Ex-smoker	AC	Early	Yes	Yes	1098*	1098*
L27	67	Male	White	Ex-smoker	AC	Early	Yes	Yes	1167*	1167*
L28	47	Female	White	Ex-smoker	AC	Late	Yes	No	746	No
L29	54	Male	Black or African American	Ex-smoker	AC	Late	Yes	No	184	No
L30	67	Female	White	Ex-smoker	AC	Early	Yes	No	2640*	1289
L31	72	Male	White	Ex-smoker	SCLC	Late	Yes	Yes	No	No

L32	62	Female	White	Current Smoker	AC	Early	Yes	No	754	482
L33	48	Male	White	Ex-smoker	AC	Late	Yes	Yes	98	No
L34	68	Male	White	Ex-smoker	AC	Late	Yes	No	170	No
L35	57	Female	White	Current Smoker	AC	Early	Yes	Yes	1021*	1021*
L36	56	Male	White	Current Smoker	AC	Late	Yes	Yes	462	339
L37	53	Female	White	Current Smoker	AC	Late	Yes	No	No	No
L38	56	Female	Black or African American	Current Smoker	AC	Early	Yes	Yes	1146*	1146*
L39	53	Female	White	Current Smoker	AC	Early	Yes	Yes	1093*	1093*
L40	53	Male	White	Current Smoker	SCC	Early	Yes	No	1099*	1099*
L41	70	Female	White	Ex-smoker	Large	Early	Yes	No	No	No
L42	63	Male	White	Current Smoker	AC	Early	Yes	Yes	1042*	1042*
L43	62	Male	White	Ex-smoker	SCC	Early	Yes	No	No	No
L44	73	Female	White	Ex-smoker	AC	Early	Yes	Yes	1010*	1010*
L45	58	Male	White	Ex-smoker	AC	Late	Yes	Yes	1131*	1131*
L46	41	Male	Asian	Ex-smoker	AC	Early	Yes	Yes	No	No
L47	60	Female	White	Never smoked	AC	Early	Yes	No	4431*	395
L48	60	Female	White	Never smoked	AC	Early	Yes	No	No	No
L49	74	Female	Black or African American	Ex-smoker	SCC	Early	Yes	No	989*	989*
L50	73	Female	White	Ex-smoker	AC	Early	Yes	No	987*	987*
L51	58	Female	White	Ex-smoker	SCC	Early	Yes	No	536	No
L52	52	Female	White	Ex-smoker	SCC	Early	Yes	Yes	1084	406
L53	63	Male	White	Ex-smoker	SCC	Late	Yes	No	252	116
L54	52	Female	White	Ex-smoker	AC	Late	Yes	Yes	445	No
L55	76	Female	White	Ex-smoker	SCC	Early	Yes	Yes	962*	962*
L56	50	Female	White	Current Smoker	AC	Early	Yes	Yes	1823*	827
L57	53	Female	White	Ex-smoker	AC	Late	Yes	No	1627	1168
L58	42	Male	White	Current Smoker	AC	Late	Yes	Yes	206	No
L59	66	Male	White	Current Smoker	AC	Late	Yes	Yes	1529	989
L60	73	Female	White	Current Smoker	SCC	Late	Yes	No	No	No
L61	52	Female	Black or African American	Ex-smoker	SCLC	Early	Yes	Yes	439	102
L62	73	Male	White	Ex-smoker	SCC	Late	Yes	Yes	250	194

L63	55	Male	White	Current Smoker	SCC	Late	Yes	Yes	No	No
L64	69	Female	Black or African American	Current Smoker	SCC	Late	Yes	No	2764	720
L65	66	Female	White	Current Smoker	SCLC	Early	Yes	Yes	No	No
L66	61	Male	White	Current Smoker	SCC	Late	Yes	No	689	267
L67	78	Male	White	Ex-smoker	SCC	Early	Yes	Yes	521	No
L68	44	Male	White	Current Smoker	AC	Late	Yes	No	718	282
L69	47	Male	Black or African American	Current Smoker	SCC	Early	Yes	Yes	3260	2995
L70	47	Female	White	Current Smoker	AC	Early	Yes	Yes	3248*	3248*
L71	70	Female	White	Ex-smoker	SCLC	Early	Yes	Yes	No	No
L72	48	Male	Black or African American	Current Smoker	NOS	Late	Yes	No	128	No
L73	71	Male	Black or African American	Current Smoker	SCC	Early	Yes	Yes	No	No
L74	75	Male	White	Ex-smoker	Large	Early	Yes	No	347	176
L75	49	Male	White	Current Smoker	SCLC	Early	Yes	Yes	256	196
L76	74	Male	White	Ex-smoker	SCC	Late	Yes	No	238	No
L77	50	Female	White	Current Smoker	SCLC	Late	Yes	Yes	275	No
L78	47	Female	White	Ex-smoker	AC	Early	Yes	Yes	447	218
L79	49	Male	White	Ex-smoker	AC	Late	Yes	Yes	1044	589
L80	75	Male	White	Ex-smoker	SCC	Late	Yes	No	No	No
L81	66	Male	Black or African American	Current Smoker	NOS	Late	Yes	No	198	No
L82	63	Male	White	Ex-smoker	SCLC	Late	Yes	Yes	380	No
L83	64	Male	Black or African American	Ex-smoker	AC	Late	Yes	Yes	476	188
L84	77	Male	White	Ex-smoker	SCC	Early	Yes	No	70	No
L85	62	Female	White	Ex-smoker	AC	Early	Yes	Yes	2035	594
L86	54	Male	White	Current Smoker	NOS	Late	Yes	No	73	No
L87	51	Female	Black or African American	Current Smoker	AC	Late	Yes	Yes	159	No
L88	55	Female	White	Current Smoker	NOS	Late	Yes	No	93	80
L89	66	Female	White	Ex-smoker	SCLC	Early	Yes	Yes	2524*	2524*

L90	65	Female	White	Current Smoker	NOS	Late	Yes	No	248	No
L91	76	Male	White	Ex-smoker	SCC	Early	Yes	No	1022	284
L92	57	Male	White	Ex-smoker	SCC	Late	Yes	Yes	495	211
L93	65	Female	White	Current Smoker	SCC	Early	Yes	Yes	2499*	2499*
L94	48	Female	White	Ex-smoker	SCLC	Early	Yes	Yes	206	162
L95	63	Female	White	Ex-smoker	AC	Late	Yes	No	105	No
L96	43	Female	Black or African American	Current Smoker	AC	Early	Yes	Yes	2580*	2580*
L97	75	Female	White	Ex-smoker	AC	Late	Yes	No	1595	1290
L98	50	Male	Black or African American	Current Smoker	AC	Early	Yes	No	1504	1348
L99	58	Male	White	Unknown	AC	Late	Yes	No	186	No
L100	62	Male	White	Current Smoker	SCC	Late	Yes	Yes	808	358
L101	43	Male	White	Current Smoker	SCLC	Early	Yes	Yes	2540*	2540*
L102	51	Female	Black or African American	Ex-smoker	AC	Early	Yes	Yes	2429*	2429*
L103	51	Male	White	Never smoked	AC	Late	Yes	No	592	315
L104	59	Female	Black or African American	Current Smoker	AC	Early	Yes	Yes	369	287
L105	61	Female	Black or African American	Current Smoker	SCC	Early	Yes	Yes	1171	630
L106	50	Male	White	Ex-smoker	AC	Late	Yes	Yes	103	No
L107	44	Female	White	Ex-smoker	AC	Late	Yes	No	2166	658
L108	59	Female	White	Current Smoker	AC	Early	Yes	No	2335*	1685
L109	58	Male	White	Current Smoker	AC	Late	Yes	Yes	282	No
L110	65	Female	White	Ex-smoker	SCC	Late	Yes	Yes	109	No
L111	65	Male	White	Never smoked	AC	Late	Yes	No	3664	1808
L112	47	Male	White	Ex-smoker	SCC	Late	Yes	No	597	336
L113	52	Female	White	Ex-smoker	SCLC	Late	Yes	No	437	303
L114	51	Male	White	Ex-smoker	SCC	Late	Yes	No	177	141
L115	49	Female	White	Current Smoker	SCC	Early	Yes	Yes	2251*	2251*
L116	43	Female	White	Current Smoker	SCLC	Late	Yes	Yes	298	158
L117	54	Male	Black or African American	Current Smoker	AC	Late	Yes	No	535	332

L118	56	Male	Black or African American	Current Smoker	SCLC	Early	Yes	No	1736	1616
L119	56	Female	White	Ex-smoker	AC	Early	Yes	Yes	1359	No
L120	49	Female	Black or African American	Ex-smoker	AC	Early	Yes	Yes	3849*	3849*
L121	57	Female	White	Current Smoker	SCC	Early	Yes	No	1918*	1918*
L122	65	Male	White	Current Smoker	AC	Early	Yes	No	224	No
L123	49	Male	White	Ex-smoker	Mix	Early	Yes	No	874	351
L124	61	Male	Black or African American	Ex-smoker	NOS	Early	Yes	No	1823*	1823*
L125	57	Female	Black or African American	Current Smoker	AC	Early	Yes	Yes	518	No
L126	49	Female	White	Current Smoker	SCC	Early	Yes	Yes	322	No
L127	53	Female	White	Current Smoker	SCC	Late	Yes	Yes	115	No
L128	67	Female	Black or African American	Ex-smoker	AC	Late	Yes	No	260	No
L129	48	Male	White	Never smoked	AC	Early	Yes	No	No	No
L130	55	Female	White	Current Smoker	NOS	Early	Yes	No	129	No
L131	60	Male	Black or African American	Ex-smoker	Large	Early	Yes	No	407	No
L132	50	Male	Black or African American	Current Smoker	AC	Late	Yes	Yes	1267	894
L133	79	Male	White	Ex-smoker	AC	Late	Yes	No	117	No
L134	55	Female	White	Current Smoker	SCC	Late	Yes	Yes	72	No
L135	61	Female	White	Current Smoker	SCLC	Early	Yes	No	336	No
L136	47	Male	White	Current Smoker	AC	Late	Yes	Yes	583	No
L137	57	Female	White	Current Smoker	AC	Late	Yes	Yes	308	222
L138	63	Female	White	Ex-smoker	SCC	Early	Yes	Yes	135	No
L139	45	Female	White	Ex-smoker	AC	Late	Yes	No	456	No
L140	66	Female	White	Ex-smoker	SCC	Late	Yes	Yes	293	No
L141	71	Male	Black or African American	Ex-smoker	SCC	Late	Yes	Yes	320	No
L142	54	Female	White	Ex-smoker	SCLC	Early	Yes	Yes	No	No
L143	62	Male	White	Unknown	SCC	Late	Yes	No	222	122

L144	49	Female	White	Current Smoker	AC	Late	Yes	Yes	143	No
L145	50	Male	White	Current Smoker	AC	Late	Yes	No	369	No
L146	44	Male	Black or African American	Current Smoker	Large	Early	Yes	No	666	501
L147	71	Male	White	Ex-smoker	SCC	Late	Yes	No	1323	1238
L148	71	Male	White	Current Smoker	AC	Early	Yes	No	439	421

Legend: AC, Adenocarcinoma; DSC, differential scanning calorimetry; Early, early–stage (stages 1–3a); Large, large cell carcinoma; Late, late-stage (stages 3b–4); Mix, Mixed type; NOS, not otherwise specified; Gly, glycosylation analysis; OS, overall survival analysis; PFS, progression-free survival analysis; SCC, squamous cell carcinoma; SCLC, small cell lung cancer; * indicates samples for whom the event (death or progression) had not occurred at the time of data collection, where the survival time indicates the end of observation for this study. For samples for which OS and PFS data were available, the time is included in the table as a day count.

Table S3. P-values for the difference in summary metrics or PCs of LC and control patients across LC type, stage, smoking status, overall and progression-free survival.

Group Comparison vs Control				
**Parameter**	**All LC patients**		**Type of LC**	
	**Unadjusted *p*-value**	**FDR[1] adjusted *p*-value**	**Unadjusted *p*-value**	**FDR[1] adjusted *p*-value**
Area	0.1779	0.3380	0.1479	0.4684
Height	0.7222	0.8884	0.9268	0.9698
Width	0.7481	0.8884	0.6513	0.9698
T_{max}	0.0101	0.0479	0.2522	0.5990
T_{FM}	0.1987	0.3432	0.7125	0.9698
Peak 1	0.6178	0.8384	0.8975	0.9698
Peak 2	0.4634	0.6773	0.9698	0.9698
Peak 3	0.0244	0.0773	0.1769	0.4802
Peak 1/2	0.9653	0.9653	0.7767	0.9698
Peak 1/3	0.3599	0.5698	0.4401	0.7819
Peak 2/3	0.0060	0.0380	0.0022	0.0418
T_{peak1}	0.0020	0.0190	0.0896	0.3792
T_{peak2}	0.1021	0.2667	0.4470	0.7819
PC1	0.9205	0.9653	0.8880	0.9698
PC2	0.0003	0.0057	0.0299	0.2841
PC3	0.1388	0.2930	0.0936	0.3792
PC4	0.0161	0.0773	0.0998	0.3792
PC5	0.7844	0.9653	0.8831	0.9698
PC6	0.1123	0.2667	0.4527	0.7819

Stage comparison vs Control								
**Parameter**	**All LC patients**		**AC**		**SCC**		**SCLC**	
	**Unadjusted p-value**	**FDR[1] adjusted p-value**	**Unadjusted p-value**	**FDR[1] adjusted p-value**	**Unadjusted p-value**	**FDR[1] adjusted p-value**	**Unadjusted p-value**	**FDR[1] adjusted p-value**
Area	0.2839	0.6335	0.1218	0.3306	0.9805	0.9805	0.2337	0.5550
Height	0.9104	0.9126	0.8443	0.9611	0.7611	0.9641	0.6485	0.7784
Width	0.6654	0.8428	0.6654	0.9611	0.5621	0.9641	0.6998	0.7784
T_{max}	0.3579	0.6335	0.7051	0.9611	0.0094	0.0893	0.5189	0.7784
T_{FM}	0.4001	0.6635	0.4091	0.8081	0.6725	0.9641	0.3722	0.7072

Peak 1	0.9049	0.9126	0.8953	0.9611	0.6397	0.9641	0.7005	0.7784
Peak 2	0.3949	0.6335	0.4253	0.8081	0.4065	0.9641	0.4933	0.7784
Peak 3	0.0562	0.2136	0.0832	0.2891	0.7421	0.9641	0.1440	0.4688
Peak 1/2	0.7726	0.9126	0.8157	0.9611	0.6046	0.9641	0.6309	0.7784
Peak 1/3	0.6398	0.8428	0.5091	0.8794	0.9312	0.9805	0.3057	0.6454
Peak 2/3	0.0014	0.0095	0.0018	0.0181	0.4245	0.9641	0.1503	0.4688
T_{peak1}	0.0015	0.0095	0.0019	0.0181	0.0144	0.0912	0.7374	0.7784
T_{peak2}	0.3208	0.6335	0.0913	0.2891	0.6591	0.9641	0.0024	0.0456
PC1	0.9126	0.9126	0.9301	0.9611	0.5762	0.9641	0.7962	0.7962
PC2	0.0001	0.0019	0.0139	0.0880	0.0087	0.0893	0.1017	0.4688
PC3	0.2475	0.6334	0.1682	0.3995	0.9653	0.9805	0.1424	0.4688
PC4	0.0382	0.1815	0.0257	0.1221	0.2503	0.9641	0.5807	0.7784
PC5	0.6435	0.8428	0.9611	0.9611	0.9202	0.9805	0.1646	0.4688
PC6	0.1954	0.6188	0.7513	0.9611	0.3649	0.9641	0.1727	0.4688

Smoking status

Parameter	All LC patients	
	Unadjusted p-value	FDR[1] adjusted p-value
Area	0.6718	0.8973
Height	0.8501	0.8973
Width	0.5282	0.8973
T_{max}	0.5804	0.8973
T_{FM}	0.5282	0.8973
Peak 1	0.9673	0.9673
Peak 2	0.4737	0.8973
Peak 3	0.1900	0.7220
Peak 1/2	0.8423	0.8973
Peak 1/3	0.7721	0.8973
Peak 2/3	0.3263	0.8973
T_{peak1}	0.0314	0.1988
T_{peak2}	0.7690	0.8973
PC1	0.7866	0.8973
PC2	0.0093	0.1739
PC3	0.4660	0.8973
PC4	0.0183	0.1739
PC5	0.0880	0.4180
PC6	0.3850	0.8973

Overall Survival

Parameter	All LC patients		AC		SCC		SCLC	
	Unadjusted p-value	FDR[1] adjusted p-value	Unadjusted p-value	FDR[1] adjusted p-value	Unadjusted p-value	FDR[1] adjusted p-value	Unadjusted p-value	FDR[1] adjusted p-value
Area	0.2684	0.5917	0.4810	0.5782	0.2808	0.9551	0.8751	0.9854
Height	0.3423	0.5917	0.4722	0.5782	0.5310	0.9551	0.7661	0.9854
Width	0.6283	0.7761	0.4233	0.5782	0.8041	0.9551	0.9136	0.9854
T_{max}	0.0214	0.1123	0.0149	0.1012	0.6569	0.9551	0.0206	0.1442
T_{FM}	0.7669	0.8476	0.8390	0.8810	0.5311	0.9551	0.9136	0.9854
Peak 1	0.3573	0.5917	0.4956	0.5782	0.5310	0.9551	0.7661	0.9854
Peak 2	0.1289	0.4512	0.0428	0.1498	0.5457	0.9551	0.9854	0.9854
Peak 3	0.6694	0.7810	0.9967	0.9967	0.8700	0.9551	0.9427	0.9854
Peak 1/2	0.4913	0.7267	0.4116	0.5782	0.7300	0.9551	0.4945	0.9854

Peak 1/3	0.9167	0.9443	0.4319	0.5782	0.3934	0.9551	0.9136	0.9854
Peak 2/3	0.0142	0.0994	0.0201	0.1012	0.4782	0.9551	0.6969	0.9854
T_{peak1}	0.0077	0.0994	0.0077	0.1012	0.5800	0.9551	0.0206	0.1442
T_{peak1} IQR	0.5839	0.7663	0.1842	0.5526	0.6226	0.9551	0.2536	0.9854
T_{peak2}	0.3663	0.5917	0.3950	0.5782	0.5314	0.9551	0.0016	0.0336
T_{peak2} IQR	0.0739	0.3103	0.0155	0.1012	0.9501	0.9551	0.8345	0.9854
PC1	0.5191	0.7267	0.2983	0.5782	0.7618	0.9551	0.4945	0.9854
PC2	0.0133	0.0994	0.0241	0.1012	0.8792	0.9551	0.5324	0.9854
PC3	0.1694	0.5082	0.4422	0.5782	0.2419	0.9551	0.8751	0.9854
PC4	0.2875	0.5917	0.8008	0.8810	0.1260	0.9551	0.9428	0.9854
PC5	0.9443	0.9443	0.3228	0.5782	0.7782	0.9551	0.1919	0.9854
PC6	0.2397	0.5917	0.3763	0.5782	0.9551	0.9551	0.3129	0.9854
**Progression-free survival**								
	**All LC patients**		**AC**		**SCC**		**SCLC**	
**Parameter**	**Unadjusted p-value**	**FDR[1] adjusted p-value**	**Unadjusted p-value**	**FDR[1] adjusted p-value**	**Unadjusted p-value**	**FDR[1] adjusted p-value**	**Unadjusted p-value**	**FDR[1] adjusted p-value**
Area	0.1026	0.4309	0.2315	0.5402	0.1806	0.9001	0.4000	0.8357
Height	0.3770	0.6863	0.3583	0.6840	0.6348	0.9435	0.7163	0.8357
Width	0.8829	0.9144	0.5488	0.7083	0.6046	0.9435	0.6547	0.8357
T_{max}	0.0014	0.0098	0.0863	0.4652	0.0747	0.7844	0.1005	0.5276
T_{FM}	0.6616	0.8363	0.5734	0.7083	0.8655	0.9435	0.6547	0.8357
Peak 1	0.4214	0.6864	0.4106	0.7083	0.6348	0.9435	0.7163	0.8357
Peak 2	0.1501	0.4503	0.0730	0.4652	0.7623	0.9435	0.8715	0.8860
Peak 3	0.1269	0.4442	0.4835	0.7083	0.5166	0.9435	0.5487	0.8357
Peak 1/2	0.8477	0.9144	0.7552	0.7930	0.8986	0.9435	0.8860	0.8860
Peak 1/3	0.6770	0.8363	0.6132	0.7121	0.3318	0.9435	0.6547	0.8357
Peak 2/3	0.2040	0.5355	0.1329	0.4652	0.8495	0.9435	0.5487	0.8357
T_{peak1}	0.0004	0.0084	0.1231	0.4652	0.0348	0.7308	0.1005	0.5276
T_{peak1} IQR	0.5340	0.7476	0.3581	0.6840	0.7968	0.9435	0.6997	0.8357
T_{peak2}	0.2742	0.6113	0.5668	0.7083	0.6543	0.9435	0.3530	0.8357
T_{peak2} IQR	0.4249	0.6864	0.2259	0.5402	0.8031	0.9435	0.0082	0.1722
PC1	0.2911	0.6113	0.2110	0.5402	0.6348	0.9435	0.8860	0.8860
PC2	0.0008	0.0084	0.0932	0.4652	0.1603	0.9009	0.2125	0.8357
PC3	0.0638	0.3350	0.1280	0.4652	0.2939	0.9435	0.4000	0.8357
PC4	0.7608	0.8776	0.6443	0.7121	0.9538	0.9538	0.5186	0.8357
PC5	0.4786	0.7179	0.8388	0.8388	0.8319	0.9435	0.0266	0.2793
PC6	0.9144	0.9144	0.4906	0.7083	0.2145	0.9009	0.3717	0.8357

Legend: AC, Adenocarcinoma; [1] FDR, false discovery rate; IQR, interquartile range [Q1, Q3]; LC, lung cancer; PC, principal component; SCC, squamous cell carcinoma; SCLC, small cell lung cancer.

Table S4. Associations between summary metrics / PCs and the clinical group. Summary metrics / PCs were dichotomized based on either the median value or interquartile range (IQR) calculated for control subjects.

**Parameter**	**Range**	**Control (n = 51)**	**LC (n = 148)**	**Unadjusted *p*-value**	**FDR[1] adjusted *p*-value**
Area	< median	25 (49.0%)	70 (47.3%)	0.8718	0.9202
	≥ median	26 (51.0 %)	78 (52.7%)		
	Outside IQR	26 (51.0 %)	81 (54.7%)	0.7449	0.9999
	Within IQR	25 (49.0%)	67 (45.3)		
Height	< median	25 (49.0%)	63 (42.6%)	0.5135	0.8908
	≥ median	26 (51.0 %)	85 (57.4%)		

	Outside IQR<br>Within IQR	26 (51.0 %)<br>25 (49.0%)	64 (43.2%)<br>84 (56.8%)	0.4150	0.8166
Width	< median<br>≥ median	24 (47.1%)<br>27 (52.9 %)	70 (47.3%)<br>78 (52.7%)	0.9999	0.9999
	Outside IQR<br>Within IQR	27 (52.9 %)<br>24 (47.1%)	84 (56.8%)<br>64 (43.2%)	0.7439	0.9999
T_{FM}	< median<br>≥ median	25 (49.0%)<br>26 (51.0 %)	66 (44.6%)<br>82 (55.4%)	0.6268	0.9202
	Outside IQR<br>Within IQR	26 (51.0 %)<br>25 (49.0%)	75 (50.7%)<br>73 (49.3%)	0.9999	0.9999
T_{max}	Outside IQR<br>Within IQR	25 (49.0 %)<br>26 (510%)	84 (56.8%)<br>64 (43.2%)	0.5157	0.8165
T_{peak1}	Outside IQR<br>Within IQR	26 (51.0 %)<br>25 (49.0%)	86 (58.1%)<br>62 (41.9)	0.4152	0.8165
T_{peak2}	< median<br>≥ median	25 (49.0%)<br>26 (51.0 %)	53 (35.8%)<br>95 (64.2%)	0.1000	0.2714
Peak 1	< median<br>≥ median	25 (49.0%)<br>26 (51.0 %)	63 (42.6%)<br>85 (57.4%)	0.5135	0.8908
	Outside IQR<br>Within IQR	26 (51.0 %)<br>25 (49.0%)	65 (43.9%)<br>83 (56.1%)	0.4176	0.8165
Peak 2	< median<br>≥ median	25 (49.0%)<br>26 (51.0 %)	67 (45.3%)<br>81 (54.7%)	0.7449	0.9202
	Outside IQR<br>Within IQR	26 (51.0 %)<br>25 (49.0%)	77 (52.0%)<br>71 (48.0%)	0.9999	0.9999
Peak 3	< median<br>≥ median	25 (49.0%)<br>26 (51.0 %)	54 (36.5%)<br>94 (63.5%)	0.1360	0.3230
	Outside IQR<br>Within IQR	26 (51.0 %)<br>25 (49.0%)	84 (56.8%)<br>64 (43.2%)	0.5157	0.8165
Peak 1/2	< median<br>≥ median	25 (49.0%)<br>26 (51.0 %)	64 (43.2%)<br>84 (56.8%)	0.5157	0.8908
	Outside IQR<br>Within IQR	26 (51.0 %)<br>25 (49.0%)	75 (50.7%)<br>73 (49.3%)	0.9999	0.9999
Peak 1/3	< median<br>≥ median	25 (49.0%)<br>26 (51.0 %)	70 (47.3%)<br>78 (52.7%)	0.8718	0.9202
	Outside IQR<br>Within IQR	26 (51.0 %)<br>25 (49.0%)	87 (58.8%)<br>61 (41.2%)	0.4127	0.8165
Peak 2/3	< median<br>≥ median	25 (49.0%)<br>26 (51.0 %)	95 (64.2%)<br>53 (35.8%)	0.0682	0.2160
	Outside IQR<br>Within IQR	26 (51.0 %)<br>25 (49.0%)	75 (50.7%)<br>73 (49.3%)	0.9999	0.9999
PC1	< median<br>≥ median	25 (49.0%)<br>26 (51.0 %)	70 (47.3%)<br>78 (52.7%)	0.8718	0.9202
	Outside IQR<br>Within IQR	26 (51.0 %)<br>25 (49.0%)	73 (49.3%)<br>75 (50.7)	0.8720	0.9999
PC2	Outside IQR<br>Within IQR	26 (51.0 %)<br>25 (49.0%)	84 (56.8%)<br>64 (43.2)	0.5157	0.8165
PC3	< median<br>≥ median	25 (49.0%)<br>26 (51.0 %)	78 (52.7%)<br>70 (47.3%)	0.7456	0.9202
	Outside IQR<br>Within IQR	26 (51.0 %)<br>25 (49.0%)	94 (65.5%)<br>54 (34.5)	0.1360	0.6460
PC4	< median<br>≥ median	25 (49.0%)<br>26 (51.0 %)	50 (33.8%)<br>98 (66.2%)	0.0654	0.2160

	Outside IQR<br>Within IQR	26 (51.0 %)<br>25 (49.0%)	97 (64.9%)<br>51 (35.1%)	0.0689	0.4364
PC5	< median<br>≥ median	25 (49.0%)<br>26 (51.0 %)	74 (50.0%)<br>74 (50.0%)	0.7471	0.9202
PC6	< median<br>≥ median	25 (49.0%)<br>26 (51.0 %)	96 (64.9%)<br>52 (35.1%)	0.0667	0.2160
	Outside IQR<br>Within IQR	26 (51.0 %)<br>25 (49.0%)	92 (62.2%)<br>56 (37.8%)	0.1872	0.7114

Legend: [1]FDR, false discovery rate; IQR, interquartile range [Q1, Q3]; LC, lung cancer; PC, principal component.

Table S5. Summary of the *p*-values for repeated measures ANOVA and Tukey's multiple comparisons. Tests evaluate differences between clinical groups using mass spectrometry glycosylation data of peptides grouped according to protein function.

IMMUNOGLOBULINS

**ANOVA summary**	Unadjusted *p*-value	FDR[1] adjusted *p*-value
	<0.0001	0.0002

Tukey's multiple comparison tests

		Control	AC		SCC		SCLC	
			Early	Late	Early	Late	Lim	Ext
Control		X	0.0026	ns	<0.0001	ns	<0.0001	ns
AC	Early	0.0026	X	ns	ns	<0.0001	0.0025	ns
	Late	ns	ns	X	0.0151	0.0288	0.0003	ns
SCC	Early	<0.0001	ns	0.0151	X	<0.0001	ns	ns
	Late	ns	<0.0001	0.0288	<0.0001	X	<0.0001	0.0338
SCLC	Lim	<0.0001	0.0025	0.0003	ns	<0.0001	X	ns
	Ext	ns	ns	ns	ns	0.0338	ns	X

COMPLEMENT C3

**ANOVA summary**	Unadjusted *p*-value	FDR[1] adjusted *p*-value
	0.1028	0.1308

Tukey's multiple comparison tests

		Control	AC		SCC		SCLC	
			Early	Late	Early	Late	Lim	Ext
Control		X	ns	ns	ns	0.0205	0.0005	0.0004
AC	Early	ns	X	ns	ns	ns	ns	ns
	Late	ns	ns	X	ns	ns	ns	ns
SCC	Early	ns	ns	ns	X	ns	ns	0.0454
	Late	0.0205	ns	ns	ns	X	ns	ns
SCLC	Lim	0.0005	ns	ns	ns	ns	X	ns
	Ext	0.0004	ns	ns	0.0454	ns	ns	X

COMPLEMENT C4

**ANOVA summary**	Unadjusted *p*-value	FDR[1] adjusted *p*-value
	0.1358	0.1584

Tukey's multiple comparison tests

		Control	AC		SCC		SCLC	
			Early	Late	Early	Late	Lim	Ext
Control		X	ns	ns	ns	ns	ns	ns
AC	Early	ns	X	ns	ns	ns	ns	ns
	Late	ns	ns	X	ns	ns	ns	ns
SCC	Early	ns	ns	ns	X	ns	ns	ns
	Late	ns	ns	ns	ns	X	ns	ns
SCLC	Lim	ns	ns	ns	ns	ns	X	ns

	Ext	ns	ns	ns	ns	ns	ns	X
**ALPHA-2-MACROGLOBULIN**								
**ANOVA summary**		Unadjusted *p*-value				FDR[1] adjusted *p*-value		
		<0.0001				0.0002		
**Tukey’s multiple comparison tests**								
		Control	AC		SCC		SCLC	
			Early	Late	Early	Late	Lim	Ext
Control		X	ns	0.0147	ns	ns	0.0109	0.0250
AC	Early	ns	X	0.0143	ns	ns	ns	0.0464
	Late	0.0147	0.0143	X	0.0004	ns	<0.0001	0.0002
SCC	Early	ns	ns	0.0004	X	0.0300	ns	ns
	Late	ns	ns	ns	0.0300	X	0.0005	0.0012
SCLC	Lim	0.0109	ns	<0.0001	ns	0.0005	X	ns
	Ext	0.0250	0.0464	0.0002	ns	0.0120	ns	X
**BETA-2 GLYCOPROTEIN 1**								
**ANOVA summary**		Unadjusted *p*-value				FDR[1] adjusted *p*-value		
		0.0019				0.0030		
**Tukey’s multiple comparison tests**								
		Control	AC		SCC		SCLC	
			Early	Late	Early	Late	Lim	Ext
Control		X	ns	ns	0.0049	ns	ns	ns
AC	Early	ns	X	ns	ns	ns	ns	ns
	Late	ns	ns	X	0.0012	ns	ns	ns
SCC	Early	0.0049	ns	0.0012	X	ns	ns	ns
	Late	ns	ns	ns	ns	X	ns	ns
SCLC	Lim	ns	ns	ns	ns	ns	X	ns
	Ext	ns	ns	ns	ns	ns	ns	X
**HAPTOGLOBIN**								
**ANOVA summary**		Unadjusted *p*-value				FDR[1] adjusted *p*-value		
		<0.0001				0.0002		
**Tukey’s multiple comparison tests**								
		Control	AC		SCC		SCLC	
			Early	Late	Early	Late	Lim	Ext
Control		X	<0.0001	<0.0001	<0.0001	<0.0001	<0.0001	0.0005
AC	Early	<0.0001	X	ns	ns	ns	ns	ns
	Late	<0.0001	ns	X	ns	ns	ns	ns
SCC	Early	<0.0001	ns	ns	X	ns	ns	ns
	Late	<0.0001	ns	ns	ns	X	ns	ns
SCLC	Lim	<0.0001	ns	ns	ns	ns	X	ns
	Ext	0.0005	ns	ns	ns	ns	ns	X
**ALBUMIN**								
**ANOVA summary**		Unadjusted *p*-value				FDR[1] adjusted *p*-value		
		<0.0001				0.0002		
**Tukey’s multiple comparison tests**								
		Control	AC		SCC		SCLC	
			Early	Late	Early	Late	Lim	Ext
Control		X	ns	0.0059	<0.0001	<0.0001	0.0002	<0.0001
AC	Early	ns	X	ns	0.0056	ns	ns	0.0018
	Late	0.0059	ns	X	ns	ns	ns	0.0006
SCC	Early	<0.0001	0.0056	ns	X	0.0232	ns	ns
	Late	<0.0001	ns	ns	0.0232	X	ns	0.0014

SCLC	Lim	0.0002	ns	ns	ns	ns	X	0.0045
	Ext	<0.0001	0.0018	0.0006	ns	0.0014	0.0045	X

SEROTRANSFERRIN

ANOVA summary	Unadjusted *p*-value	FDR[1] adjusted *p*-value
	<0.0001	0.0002

Tukey's multiple comparison tests

		Control	AC		SCC		SCLC	
			Early	Late	Early	Late	Lim	Ext
Control		X	0.0287	0.0018	<0.0001	<0.0001	0.0006	0.0450
AC	Early	0.0287	X	ns	<0.0001	0.0068	ns	ns
	Late	0.0018	ns	X	ns	ns	ns	ns
SCC	Early	<0.0001	<0.0001	ns	X	ns	ns	ns
	Late	<0.0001	0.0068	ns	ns	X	ns	0.0240
SCLC	Lim	0.0006	ns	ns	ns	ns	X	ns
	Ext	0.0450	ns	ns	ns	0.0240	ns	X

HEMOPEXIN

ANOVA summary	Unadjusted *p*-value	FDR[1] adjusted *p*-value
	0.1528	0.1646

Tukey's multiple comparison tests

		Control	AC		SCC		SCLC	
			Early	Late	Early	Late	Lim	Ext
Control		X	ns	0.0093	ns	0.0482	0.0168	ns
AC	Early	ns	X	0.0356	ns	ns	0.0140	ns
	Late	0.0093	0.0356	X	ns	ns	ns	ns
SCC	Early	ns	ns	ns	X	ns	ns	ns
	Late	0.0482	ns	ns	ns	X	ns	ns
SCLC	Lim	0.0168	0.0140	ns	ns	ns	X	ns
	Ext	ns	ns	ns	ns	ns	ns	X

APOLIPOPROTEIN B100

ANOVA summary	Unadjusted *p*-value	FDR[1] adjusted *p*-value
	0.0317	0.0444

Tukey's multiple comparison tests

		Control	AC		SCC		SCLC	
			Early	Late	Early	Late	Lim	Ext
Control		X	ns	ns	ns	ns	ns	ns
AC	Early	ns	X	ns	ns	ns	ns	ns
	Late	ns	ns	X	ns	ns	ns	ns
SCC	Early	ns	ns	ns	X	ns	ns	ns
	Late	ns	ns	ns	ns	X	ns	ns
SCLC	Lim	ns	ns	ns	ns	ns	X	ns
	Ext	ns	ns	ns	ns	ns	ns	X

ALPHA 1-ANTICHYMOTRYPSIN

ANOVA summary	Unadjusted *p*-value	FDR[1] adjusted *p*-value
	0.5038	0.5038

Tukey's multiple comparison tests

		Control	AC		SCC		SCLC	
			Early	Late	Early	Late	Lim	Ext
Control		X	ns	ns	ns	ns	ns	ns
AC	Early	ns	X	ns	ns	ns	ns	ns
	Late	ns	ns	X	ns	ns	ns	ns
SCC	Early	ns	ns	ns	X	ns	ns	ns

	Late	ns	ns	ns	ns	X	ns	ns
SCLC	Lim	ns	ns	ns	ns	ns	X	ns
	Ext	ns	ns	ns	ns	ns	ns	X

Legend: [1]FDR, false discovery rate; AC, adenocarcinoma; Early, early-stage (stages 1–3a); Ext, extensive (late-stage); Late, late-stage (stages 3b–4); Lim, limited (early-stage); ns, not statistically significant at the 5% level; SCC, squamous cell carcinoma; SCLC, small cell lung cancer.
